# The polysaccharide chitosan facilitates the isolation of small extracellular vesicles from multiple biofluids

**DOI:** 10.1002/jev2.12138

**Published:** 2021-09-01

**Authors:** Awanit Kumar, Surendar Reddy Dhadi, Ngoc‐Nu Mai, Catherine Taylor, Jeremy W. Roy, David A. Barnett, Stephen M. Lewis, Anirban Ghosh, Rodney J. Ouellette

**Affiliations:** ^1^ Atlantic Cancer Research Institute Moncton New Brunswick Canada; ^2^ Department of Chemistry and Biochemistry Mount Allison University Sackville New Brunswick Canada; ^3^ Department of Chemistry and Biochemistry Université de Moncton Moncton New Brunswick Canada; ^4^ Beatrice Hunter Cancer Research Institute Halifax Nova Scotia Canada

**Keywords:** biofluids, chitin, chitosan, exosomes, extracellular vesicles, proteomics, sEV isolation technology

## Abstract

Several studies have demonstrated the potential uses of extracellular vesicles (EVs) for liquid biopsy‐based diagnostic tests and therapeutic applications; however, clinical use of EVs presents a challenge as many currently‐available EV isolation methods have limitations related to efficiency, purity, and complexity of the methods. Moreover, many EV isolation methods do not perform efficiently in all biofluids due to their differential physicochemical properties. Thus, there continues to be a need for novel EV isolation methods that are simple, robust, non‐toxic, and/or clinically‐amenable. Here we demonstrate a rapid and efficient method for small extracellular vesicle (sEV) isolation that uses chitosan, a linear cationic polyelectrolyte polysaccharide that exhibits biocompatibility, non‐immunogenicity, biodegradability, and low toxicity. Chitosan‐precipitated material was characterized using Western blotting, nanoparticle tracking analysis (NTA), transmission electron microscopy (TEM), and relevant proteomic‐based gene ontology analyses. We find that chitosan facilitates the isolation of sEVs from multiple biofluids, including cell culture‐conditioned media, human urine, plasma and saliva. Overall, our data support the potential for chitosan to isolate a population of sEVs from a variety of biofluids and may have the potential to be a clinically amenable sEV isolation method.

## 1 INTRODUCTION

Extracellular vesicles (EVs) are nanometer to micrometre‐sized lipid‐bilayer encapsulated entities that facilitate cell‐to‐cell communication and include exosomes and ectosomes (microparticles and microvesicles). Exosomes are formed within endosome‐derived multi‐vesicular bodies (MVBs) and are released into the extracellular space upon fusion of the MVB with the plasma membrane, whereas ectosomes are formed and released by direct budding of the plasma membrane (Théry et al., 2018; van Niel et al., 2018). Other types of EVs, such as oncosomes or apoptotic bodies, are also produced naturally and have been implicated in various pathological conditions (van Niel et al., 2018). EVs contain RNA, DNA, proteins, and metabolites derived from their cell of origin (Zaborowski et al., 2015) and have been shown to be present in several body fluids, including blood, urine, cerebrospinal fluid, bile, and breast milk (Yáñez‐Mó et al., 2015). During normal homeostatic conditions EVs are released at a basal level, but pathological conditions, such as cancer, lead to increased EV secretion and altered cargo packaging (Vasconcelos et al., 2019), which in the case of cancer can modulate the tumour microenvironment to orchestrate pathogenesis, progression, and metastasis (Maacha et al., 2019; Mondal et al., 2017). Due to the changes in EV composition caused by pathology, EVs are considered valuable materials for liquid biopsy‐based diagnostic tests. In recent years, EVs have also gained value as potential therapeutic vehicles for the treatment of cancers and other chronic diseases based on their ability to package and deliver cargo (De Toro et al., 2015).

The heterogeneity of EV populations has been described in many studies (Willms et al., 2018). EVs are primarily classified according to size (e.g. small EVs are < 100 nm or < 200 nm and medium/large EVs are > 200 nm) or density (low, medium, or high) (Théry et al., 2018). Small EVs (sEVs) sediment at ≥ 100,000 × *g* (ultracentrifugation) and include exosomes, medium EVs sediment at ≤ 20,000 × *g* and include microvesicles, whereas large EVs sediment at ≤ 2,000 × *g* and include apoptotic bodies (Mateescu et al., 2017; Théry et al., 2018). Sample preparation typically involves pre‐clearing a biofluid by either low‐speed centrifugation (2,000 × *g* to 20,000 × *g*) or filtration, which may remove most of the medium and large EVs from the sample prior to isolation (Mateescu et al., 2017). Both exosomes (30 to 150 nm) and microvesicles (100 to 1,000 nm) have a density of ∼1.1‐1.2 g/ml and are therefore difficult to separate from lipoproteins, which have a similar size and density (Brennan et al., 2020), when a biophysical property‐based method (e.g. ultracentrifugation, polymer‐mediated precipitation) is used for their isolation (Nath Neerukonda et al., 2019; Neerukonda et al., 2020). Therefore, many currently‐used EV isolation methods may co‐isolate contaminants, such as lipoproteins, or favour the isolation of a specific EV population based on the conditions used (Théry et al., 2018).

Chitin is synthesized by numerous living organisms and is one of the most abundant naturally‐occurring polymers (Elieh‐Ali‐Komi & Hamblin, 2016). Chitosan (poly [1→4]‐2‐amino‐2‐deoxy‐β‐D‐glucan) is a derivative of chitin produced by alkaline deacetylation (Elieh‐Ali‐Komi & Hamblin, 2016). Chitosan has recognized anti‐bacterial, anti‐fungal, and wound healing properties and has been demonstrated to be non‐toxic to mammalian cells (Matica et al., 2019). Chitosan is also biodegradable, biocompatible, polycationic, and does not exhibit immunogenicity (Bhatnagar & Sillanpaa, 2009; Bojar et al., 2014; Illum et al., 2001; Read et al., 2005; Vazquez et al., 2013). Accordingly, chitosan has been used for many purposes, including: gene delivery (Bowman & Leong, 2006), drug delivery (nano‐medicine) (Guo et al., 2014), cellular scaffold (Dhivya et al., 2018), cartilage tissue engineering (Rodriguez‐Vazquez et al., 2015), bone repair and regeneration (Baldrick, 2010), as well as bacterial (Amato et al., 2018), fungal (Martinez et al., 2010), and viral infection treatments (Read et al., 2005).

The pKa of chitosan varies from 6.2 to 6.5 depending on its molecular weight and degree of deacetylation (Wang et al., 2006). Chitosan is soluble at acidic pH (for example a 1% acetic acid solution with pH ∼3), but less soluble in water or common physiological buffers (Giraldo & Rivas, 2020). Acidic solutions facilitate the protonation of chitosan, thereby eliciting electrostatic repulsion of each chitosan molecule that results in solvation of this cationic polymer and non‐Newtonian fluidic behaviour (Giraldo & Rivas, 2020). By virtue of chitosan's high positive charge it may be able to interact with the negatively‐charged membranes of EVs to facilitate their isolation (Deregibus et al., 2016); moreover, chitosan may form a high‐molecular weight complex with EVs that permits EV isolation using low‐speed centrifugation.

Herein we demonstrate that chitosan facilitates the precipitation of small extracellular vesicles (sEVs) from a variety of biofluids. We compared the efficacy of chitosan‐mediated sEV precipitation with sucrose‐cushion UCF (scUCF), which was performed as a benchmark for sEV isolation. Combinations of Western blot analyses, nanoparticle tracking analysis (NTA), transmission electron microscopy (TEM), and proteome‐based gene ontology analyses were performed to quantify, characterize and validate sEVs isolated by chitosan from a variety of biofluids. Overall, our results indicate that chitosan can be used as a simple and robust sEV isolation method.

## 2 MATERIALS AND METHODS

### 2.1 Cell culture conditions and human biofluids

The human embryonic kidney cell line (HEK293) and the immortalized breast epithelial cell line MCF10A were obtained from American Type Culture Collection (ATCC) and maintained in culture according to the ATCC guidelines. For sEV collection, cells were grown in 5‐layer flasks (Corning) at a seeding density of 7 × 10^7^ cells per flask with 125 ml of DMEM media supplemented with 5% EV‐depleted FBS (commercial FBS was subjected to ultracentrifugation at 138,000 × *g* for 18 h to prepare EV‐depleted FBS). The cell‐culture conditioned media (CCM) was harvested after 3 days of culture. A two‐step centrifugation process (3,000 × *g* for 5 min followed by 17,000 × *g* for 15 min) was performed to pre‐clear the CCM from cells and cell debris, large vesicles, microvesicles and apoptotic bodies. A total of 1 μl/ml Protease Inhibitor cocktail III (EMD Millipore) and 0.1% (v/v) ProClin300 (Sigma) biocide were added to the CCM, which was then stored at 4°C.

Human plasma was obtained commercially from Innovative Research Inc. The blood was collected into K2 EDTA tubes and was processed within 1 h of collection by centrifugation at 5,000 × *g* for 15 min at 4°C to separate the plasma fraction. The plasma was stored at ‐80°C in 50 ml aliquots until needed. The plasma was shipped frozen on dry ice and stored at ‐80°C upon receipt until use. Plasma samples from male donors of the age range 24–62 years were used in this study. As plasma is a viscous biofluid, it was diluted 1:4 with 1 × PBS (for example 0.5 ml plasma diluted with 1.5 ml PBS) before pre‐clearing steps. The human saliva was obtained commercially from Lee Biosolutions. The saliva sample was pooled from male donors of age range 17–37 years at the time of collection. The donors brushed their teeth and rinse thoroughly before starting a collection and were not permitted to eat or drink anything other than water while donating. They had to wait a minimum of 30 s for the mouth to normalize if consumed water during the collection process. The saliva was frozen immediately after collection with no preservative and stored at ‐20°C without any processing. The saliva was shipped frozen on dry ice and stored at ‐80°C upon arrival. The midstream urine samples were collected from healthy volunteers with informed consent. Pooled urine samples from male donors of the age range 55–75 years old at the time of collection, were used for this study. The collected urine samples were processed by centrifugation at 650 × *g* for 10 min at room temperature. The supernatant was collected and centrifuged at 10,000 × *g* for 30 min at room temperature. This supernatant was collected and stored at ‐80°C until use without any preservative. The samples for all the biofluids were collected from non‐fasted donors at a random time of the day with no fixed schedule. Prior to sEV isolations, all the biofluids were subjected to a two‐step centrifugation process (3,000 × *g* for 5 min followed by 17,000 × *g* for 15 min) to pre‐clear from cells and cell debris, large vesicles, microvesicles, and apoptotic bodies. The study was conducted in accordance with recognized ethical guidelines, including the Declaration of Helsinki, and was approved by the Research Ethics Board of the Vitalité Health Network.

### 2.2 Sucrose cushion ultracentrifugation (scUCF)

The protocol for sEV isolation using ultracentrifugation was performed as previously described (Ghosh et al., 2014; Thery et al., 2006). In brief, 10 ml of pre‐cleared biofluids were transferred to UCF tubes (for SW40Ti Rotors, Beckman) followed by careful layering of 700 μl of sucrose cushion (0.1 μm filtered 30% sucrose solution in PBS) to the bottom of the tubes. Centrifugation was performed at 138,000 × *g* for 2 h at 4°C. The sEV‐containing sucrose cushions were carefully aspirated to new UCF tubes and diluted to 10 ml with 1 × PBS and centrifuged at 138,000 × *g* for 90 min to wash the sEVs. The sEV pellet was resuspended in 100 μl of 1 × PBS and stored at 4°C for immediate use or at ‐80°C for long‐term storage. For all the comparisons with scUCF‐based isolation, a normalized amount of scUCF‐isolated sEVs were used according to the starting volumes of biofluids used for sEV isolation with chitosan.

Further purification of scUCF‐isolated EVs prior to proteomic analyses was performed by sucrose density gradient ultracentrifugation as previously described (Arteaga‐Blanco et al., 2020). Briefly, the scUCF‐purified EV pellet from HEK‐CCM was bottom loaded on a sucrose density gradient (90%‐10%, bottom to top) and subjected to ultracentrifugation at 200,000 × *g* for 16 h. Following centrifugation six fractions (F1‐F6) were collected from the top to the bottom of the gradient and transferred into new tubes. The EVs from all the fractions were washed by adding 9 ml of 1 × PBS, and pelleted by ultracentrifugation at 138,000 × *g* for 2 h. The density gradient‐purified EVs (DG‐EVs) were subjected to Western blot analyses of canonical EV markers CD63 and CD9 to identify EV‐enriched fractions.

### 2.3 Chitosan: source and solution preparation

Chitosan from Invivogen (Catalog no. tlrl‐cht, 60–120 kDa, 75%–85% deacetylated) and Sigma (Catalog no. 42344, 141 kDa, > 75% deacetylated) were dissolved in 1% acetic acid solution as recommended by the manufacturer to prepare a stock concentration of 20 mg/ml, which was stored at 4°C; this is the acidic formulation of chitosan (pH ∼3). For experiments in which a neutral formulation of chitosan is used, the above‐described acidic formulation was neutralized to pH ∼7 with 100 mM NaOH to prepare a stock concentration of 8 mg/ml, which was stored at 4°C. Acetic acid (1%) and neutralized 1% acetic acid (with 100 mM NaOH) are termed as acidic buffer and neutral buffer, respectively, and were used as negative controls for acidic and neutralized formulations of chitosan. Chitosan‐coated (Catalog no. WHM‐G128) and Dextran‐coated (catalog no. WHM‐G013) magnetic beads were obtained commercially from Creative Diagnostics.

### 2.4 Chitosanase treatment

Chitosan was treated with chitosanase (Millipore Sigma) as per the manufacturer recommendations. One mg of the chitosan acidic formulation was incubated with 10 μl of chitosanase (approximately 1 unit) in 125 mM sodium acetate buffer (pH 5.5) for 1 h at 37°C. The digested chitosan was stored at 4°C until used. The same reaction mixture without chitosan was used as a negative control.

### 2.5 sEV precipitation with chitosan

**FIGURE 1:**
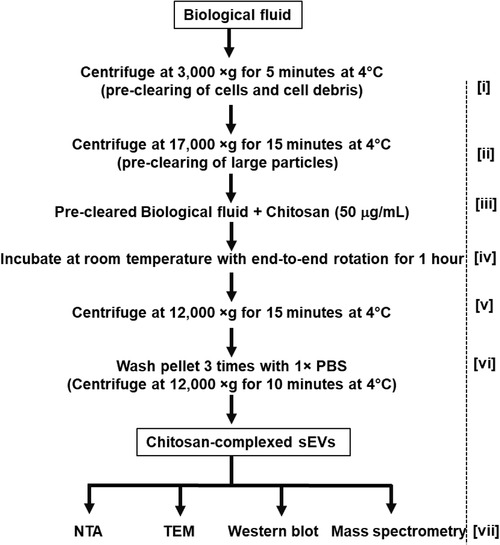
Flow chart for chitosan‐based small extracellular vesicle (sEV) isolation. Biofluids are collected and precleared in two steps; first by centrifugation at 3,000 × *g* for 5 min at 4°C [i], and a second centrifugation at 17,000 × *g* for 15 min at 4°C [ii]. Chitosan is added to a final concentration of 50 μg/ml [iii] and samples are incubated at room temperature for 1 h with end‐to‐end rotation to form chitosan‐sEV complexes [iv]. The chitosan‐sEV complexes are pelleted by centrifugation at 12,000 × *g* for 15 min at 4°C [v]. The resulting pellet of chitosan‐sEV complex is washed 3 times with 1 × PBS by centrifugation at 12,000 × *g* for 10 min at 4°C [vi]. Chitosan‐sEV complexes are used for various downstream analyses [vii], such as nanoparticle tracking analysis (NTA), transmission electron microscopy (TEM), Western blot and mass spectrometry

A flow chart that outlines the protocol is depicted in Figure 1. Chitosan was used to precipitate sEVs from pre‐cleared CCM and human biofluids. All the steps were performed in protein low‐bind Eppendorf tubes. Chitosan was added to pre‐cleared CCM/biofluids at the indicated concentrations and incubated for 1 h with end‐to‐end rotation at room temperature. Following incubation with chitosan, the tubes were centrifuged at 12,000 × *g* for 15 min at 4°C. Supernatant was discarded and the pellet was washed twice with 1 × PBS (same volume as the starting biofluid), followed by centrifugation at 12,000 × *g* for 10 min at 4°C. The pellet containing chitosan‐sEV complexes was collected and used for downstream analyses.

### 2.6 Chitosan‐magnetic bead sEV isolation

Chitosan magnetic beads were added to 1 ml of pre‐cleared CCM at various concentrations (0.5, 1, and 2 mg/ml) and incubated for 1 h at room temperature by end‐to‐end rotation. Dextran‐coated magnetic beads (concentration 2 mg/ml) were included as a negative control. After incubation the beads were collected using a magnetic stand and the supernatant was discarded; the beads were washed three times with 1 × PBS. 25 μl of 2 × Laemmli buffer was added directly to the beads, which were subjected to incubation at 95°C for 10 min prior to SDS‐PAGE and Western‐blot analyses.

### 2.7 Western blotting

The sEVs isolated by various methods were lysed in 2 × Laemmli sample loading buffer (Biorad, Cat. No. 1610737) and resolved on SDS‐PAGE (10% gels were used unless otherwise indicated). Non‐reducing conditions were used to detect exosome‐specific tetraspanins (CD63 and CD9). Bond‐Breaker TCEP Solution (Thermo Fisher Sc., #77720) was used to reduce the samples before loading on the gel. Western blotting was performed as described previously (Ghosh et al., 2014). Blots were developed using chemiluminescence substrate (Amersham ECL Prime) and documented with a Biorad Chemidoc imaging system. All the Western blot experiments were repeated at least three times. The antibodies CD63 (sc‐5275), CD9 (sc‐59140), HSC70 (sc‐7298), apolipoprotein A1 (APOA1, sc‐376818), apolipoprotein (APOB, sc‐13538), tetranectin (TETN, sc‐376940), serum amyloid A (SAA, sc‐59679), and Tamm‐Horsfall protein (THP, sc‐271022), were purchased from Santa Cruz Biotechnology. Flotillin‐1 (FLOT1, #186345) antibody was purchased from Cell Signalling Technology. H‐ficolin (FCN3, #H00008547‐A01) antibody was purchased from Abnova. Calnexin (CANX, #ab22595) antibody was purchased from Abcam. Peroxidase‐conjugated secondary antibodies (anti‐mouse IgG, Cat. No. 115‐035‐003 and anti‐rabbit IgG, Cat. No. 111‐035‐003) were obtained from Jackson Immunoresearch laboratories. Final working dilutions for primary and secondary antibodies were 1:1,000 and 1:10,000 respectively.

### 2.8 Nanoparticle tracking analysis (NTA)

NTA was performed using an NS300 instrument equipped with a 405 nm laser and software version 3.3 (Malvern instruments). sEVs were released from chitosan‐sEV complexes by adding 100 μl of 2 M NaCl. The pellets were vigorously vortexed for 1 min, incubated overnight with shaking (600 rpm) at room temperature, followed by centrifugation at 17,000 × *g* for 15 min at 4°C. The supernatant containing dispersed sEVs was carefully aspirated into a new tube. The dispersed sEVs were diluted 1:20 in 0.1 μm filtered water to bring the concentration into the recommended range for NTA. Each sample was recorded five times for 60 s and analysed in auto mode using NTA software (v. 3.3) with gain of 512 and shutter at 1,300. Size distribution data were analysed by normalizing the concentration of particles of different diameters with bin widths of 1 nm and then taking the average of each measurement. The resulting data were normalized and presented as particles per millilitre of original volume CCM/biofluids ± standard error of the mean (SEM).

### 2.9 Electron microscopy

Transmission electron microscopy (TEM) was performed according to a previously‐published method (Ghosh et al., 2014). In brief, sEVs were eluted from the chitosan‐sEV complexes as described above. The 2 M NaCl solution containing the sEVs was desalted by buffer exchange using 3 kDa spin‐filters (Nanosep columns from Pall Corporation). The desalted sEVs were deposited onto formvar/silicone monoxide coated 200 mesh copper grids (Electron microscopy Sciences) for 2–3 min, followed by fixation with 3.7% formalin and washed twice with water. The samples were contrasted with 2% Uranyl Acetate (w/v). All the solutions used were 100 kDa filtered to avoid particulate deposits on the grids. The dried grids were viewed using a JEOL 6400 electron microscope.

### 2.10 Mass spectrometry and gene ontology

Mass Spectrometry (MS) was performed according to our previously‐published protocol (Ghosh et al., 2014). The peptide mixtures eluted from the gel slices were separated by gradient reversed‐phase nano‐liquid chromatography for 70 min. LC‐MS/MS data was searched against the human UniProt database using the SequestHT algorithm in Proteome Discoverer 2.0 for protein prediction and identification. The samples were run in triplicate and the proteomic lists were prepared with proteins that appeared in at least two out of three replicates. The protein lists were subjected to FunRich (http://www.funrich.org) and PantherDB (http://geneontology.org) for gene ontology (GO) to identify the best match with cellular components.

### 2.11 EV‐TRACK

We have submitted all relevant data of our experiments to the EV‐TRACK knowledgebase (EV‐TRACK ID: EV210144) (Van Deun et al., 2017).

## 3 RESULTS

### 3.1 Chitosan isolates sEVs from cell culture conditioned media

Chitosan has a high positive‐charge density (Bellich et al., 2016) that may permit it to interact with the negatively‐charged membranes of EVs (Deregibus et al., 2016), thereby forming a high molecular weight chitosan‐EV complex that could be isolated by low‐speed centrifugation. We therefore chose to determine if the addition of chitosan to cell culture conditioned media (CCM) could facilitate the isolation of sEVs by low‐speed centrifugation. CCM harvested from HEK‐293 cells was subjected to preclearing by a two‐step centrifugation process (3,000 × *g* and 17,000 × *g*) to remove cells, cell debris, apoptotic bodies, and large vesicles. We first determined the optimal final concentration of chitosan to use for our experiments by incubating 2 ml of precleared CCM with increasing amounts of 60–120 kDa chitosan (Invivogen), following the isolation protocol outlined in Figure 1 and the Materials & Methods section. Two formulations of chitosan were used for this study: an acidic formulation in which chitosan is solubilized in 1% acetic acid, and a neutral formulation in which the chitosan that has been solubilized in 1% acetic acid is adjusted to neutral pH (∼pH 7.0) by the addition of 100 mM NaOH. We observed a dose‐dependent increase in signal for canonical EV markers by Western blot analysis for material isolated by the addition of increasing concentrations (10 μg/ml to 200 μg/ml) of the acidic formulation of chitosan (Supplemental Figure S1). Specifically, we could detect the isolation of canonical EV markers by Western blot at a minimum final concentration of 25 μg/ml for the acidic formulation of chitosan, with optimal signal for canonical EV markers achieved by a final concentration of 50 μg/ml (Supplemental Figure S1). In contrast, we were only able to detect a weak signal for some canonical EV markers (CD9 and CD63) for the addition of up to 200 μg/ml of the neutral formulation of chitosan (Supplemental Figure S1). Based on these results we chose to use a final concentration of 50 μg/ml of the acidic formulation of 60–120 kDa chitosan for the majority of our experiments.

**FIGURE 2:**
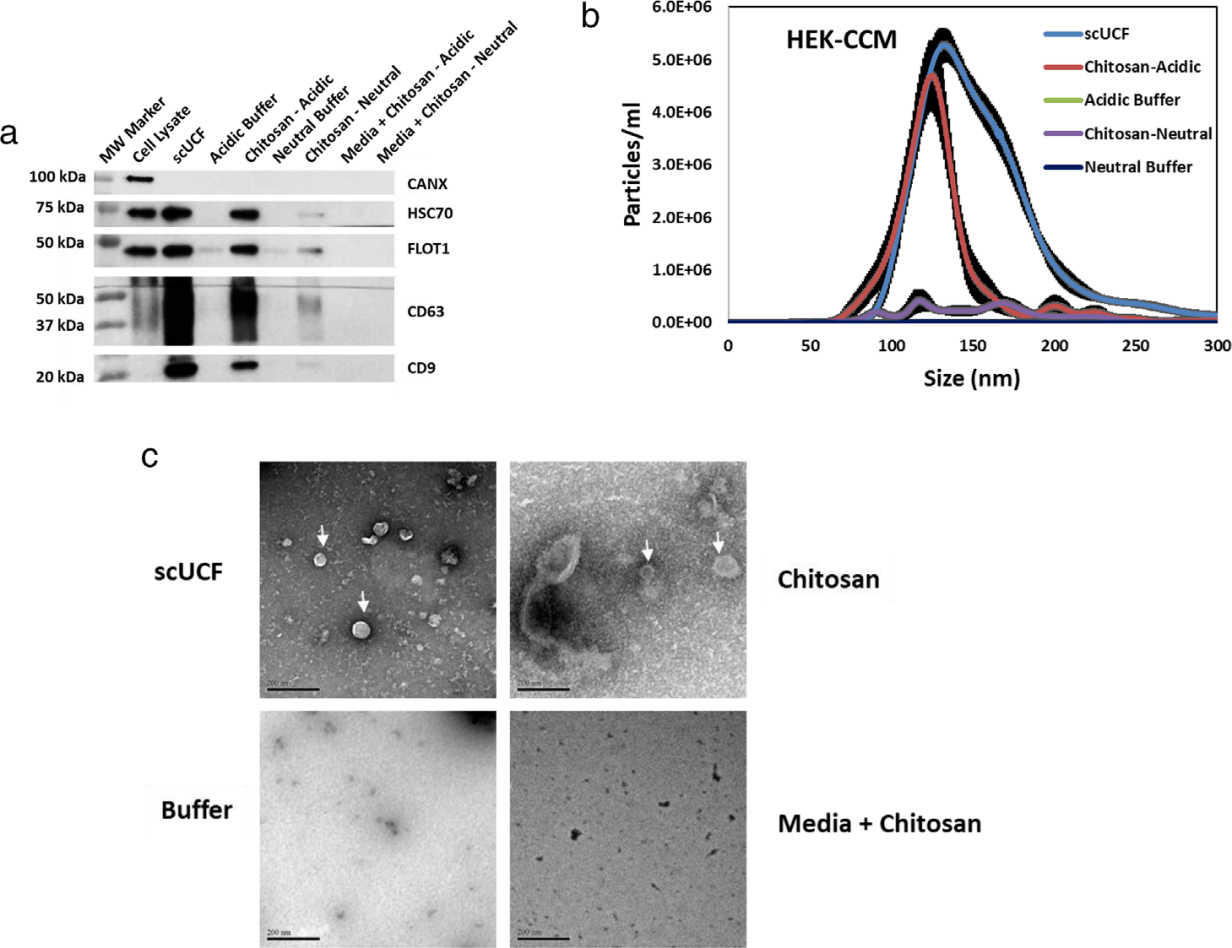
Chitosan‐mediated sEV isolation from HEK‐293 conditioned culture media (CCM). (a) Western blot analyses of the canonical EV markers CD63, CD9, HSC70, and FLOT1 for material isolated by the addition of acidic or neutral formulations of 60–120 kDa chitosan at a final concentration of 50 μg/ml to 2 ml of CCM. sEVs isolated using scUCF from 2 ml of CCM were used as a positive control and the acidic or neutral buffers in which chitosan was dissolved were included as negative controls. Non‐conditioned media (Media) was also used to evaluate whether any sEV artefacts are present within the chitosan preparation. Blots were also probed for CANX, which is absent from sEVs; total cell lysate from MCF‐10A cells was used as a positive control for CANX. (b) NTA analysis of sEVs isolated from 2 ml of HEK‐293 CCM by scUCF, the acidic formulation of chitosan, the neutral formulation of chitosan, and buffer controls. Particle concentration (particles/ml) is plotted against size (nm) of particles. The mean and the standard error of the mean (n = 3) are shown. (c) TEM images of sEVs isolated by scUCF, chitosan (acidic formulation), and buffer from HEK‐293 CCM. Non‐conditioned media incubated with the acidic formulation of chitosan was also used to confirm that chitosan does not aggregate into sEV‐sized particles. Scale bars represent 200 nm. Membrane‐bound structures consistent with sEVs are indicated with white arrows

To confirm that we are able to isolate sEVs by the addition of chitosan to CCM, we added chitosan (acidic or neutral formulation) to 2 ml of precleared CCM to a final concentration of 50 μg/ml, followed by incubation and low‐speed centrifugation to precipitate chitosan‐sEV complexes (Figure 1). The isolated material was first separated by SDS‐PAGE and subjected to Western blot analyses for the canonical EV markers heat shock cognate 71kda protein (HSC70), flotillin‐1 (FLOT1), and the tetraspanins CD63 and CD9 (Théry et al., 2018), as well as the non‐EV marker calnexin (CANX), which is an ER‐associated chaperone not found in sEVs (Tucher et al., 2018). We included sEV isolation from an equivalent volume of CCM by sucrose‐cushion ultracentrifugation (scUCF) as a positive control and the addition of equivalent volumes of the 1% acetic acid solution or neutralized solution in which chitosan is solubilized as negative controls (‘Acidic Buffer’ and ‘Neutral Buffer’). We were able to detect appreciable signal for the canonical EV markers HSC70, FLOT1, CD63, and CD9 in the material isolated by scUCF and by the addition of the acidic formulation of chitosan (Figure 2a); however, we did not detect any significant signal for these proteins in the material isolated by the addition of the buffers. A weak signal was observed for canonical EV markers for material isolated by the addition of the neutral formulation of chitosan, confirming our previous finding that the neutral formulation of chitosan has weak activity for sEV marker isolation (Supplemental Figure S1). Importantly, neither the material isolated by scUCF nor the acidic and neutral formulations of chitosan showed any signal for CANX (Figure 2a), indicating both scUCF‐ and chitosan‐isolated materials are devoid of cellular contaminants. We also ruled out the possibility that the chitosan preparation could be contaminated with canonical EV marker proteins by incubating non‐conditioned culture media (‘Media’) with 50 μg/ml chitosan. We found that there is no signal for canonical EV markers for the material isolated from non‐conditioned media (Figure 2a), indicating that our preparations of chitosan are not contaminated with canonical EV proteins. Together these results indicate that chitosan isolates material that contains canonical EV proteins, suggesting that chitosan can isolate sEVs from CCM.

We next characterized the size distribution and particle concentration of the isolated material by performing nanoparticle tracking analysis (NTA). Material, including extracellular vesicles, was isolated from 2 ml of CCM by scUCF, the addition of acidic or neutral formulations of chitosan to a final concentration of 50 μg/ml, or the addition of buffer controls. The chitosan‐isolated material must first be dispersed and released into solution before NTA; we therefore used a high ionic‐strength salt solution (2 M NaCl) as described in the Materials & Methods section to disrupt the putative chitosan‐sEV complexes. A similar high ionic elution approach was previously used to release sEVs captured by heparin (Balaj et al., 2015). The eluted material was diluted with distilled water to enumerate size distribution and relative concentrations using NTA. We found that the size range of particles that were high salt‐eluted from the putative chitosan‐sEV complexes is ∼80 nm to ∼170 nm, with an average size of ∼133 nm and mode size of ∼125 nm, which differs slightly from scUCF‐isolated sEVs, which have a wider size distribution of ∼90 nm to ∼200 nm, with an average size of ∼165 nm and mode size of ∼133 nm (Figure 2b). Both the acidic formulation of chitosan and scUCF isolated particles are consistent in size with sEVs (Théry et al., 2018). The average concentration of particles isolated by the acidic formulation of chitosan (2.02 × 10^8^ particles/ml) was comparable to the average concentration of particles isolated by scUCF (4.08 × 10^8^ particles/ml; Figure 2b). In contrast, the neutral formulation of chitosan and the buffer controls did not isolate an appreciable number of particles, which precluded size analysis by NTA (Figure 2b). These results show that chitosan isolates material that has a size range characteristic of sEVs, and that the number of particles isolated is comparable to scUCF‐mediated sEV isolation (Supplementary Table S1).

We next assayed the morphological characteristics of the chitosan‐isolated material by performing transmission electron microscopy (TEM; Figure 2c and Supplemental Figure S2). Material was isolated from 2 ml of CCM by adding the acidic formulation of chitosan (hereafter referred to as ‘chitosan’) to a final concentration of 50 μg/ml. For comparison, we also isolated sEVs from 2 ml of CCM by scUCF and included the acidic buffer as a negative control. A 2 M NaCl solution was used to elute material from the putative chitosan‐sEV complexes as described above for NTA. The eluted material was buffer exchanged using 3 kDa cut‐off spin filters for desalting prior to fixing and counterstaining for the preparation of TEM grids. TEM analysis showed round intact structures with double (or multiple) membrane folds, which are characteristic features of sEVs (Lobb et al., 2015), for both the scUCF‐isolated material and the chitosan‐isolated material (Figure 2c, upper panels). Material isolated by the addition of an equivalent volume of the acidic buffer did not display any similar structures or the presence of any membrane‐bound features (Figure 2c, lower left panel). In order to confirm that chitosan itself or chitosan aggregates do not produce structures of similar morphology, we also prepared TEM grids from non‐conditioned culture media (’Media’) that was incubated with a final concentration of 50 μg/ml chitosan. As shown in Figure 2c (lower right panel), no sEV‐like structures were observed in the chitosan‐isolated material from non‐conditioned media, indicating that the observed structures are not due to aggregation of chitosan itself. These results show that the material isolated by chitosan has the morphological characteristics of sEVs. Altogether the above‐described results indicate that the addition of 60–120 kDa chitosan to CCM at a final concentration of 50 μg/ml permits the isolation of sEVs by low‐speed centrifugation.

**FIGURE 3:**
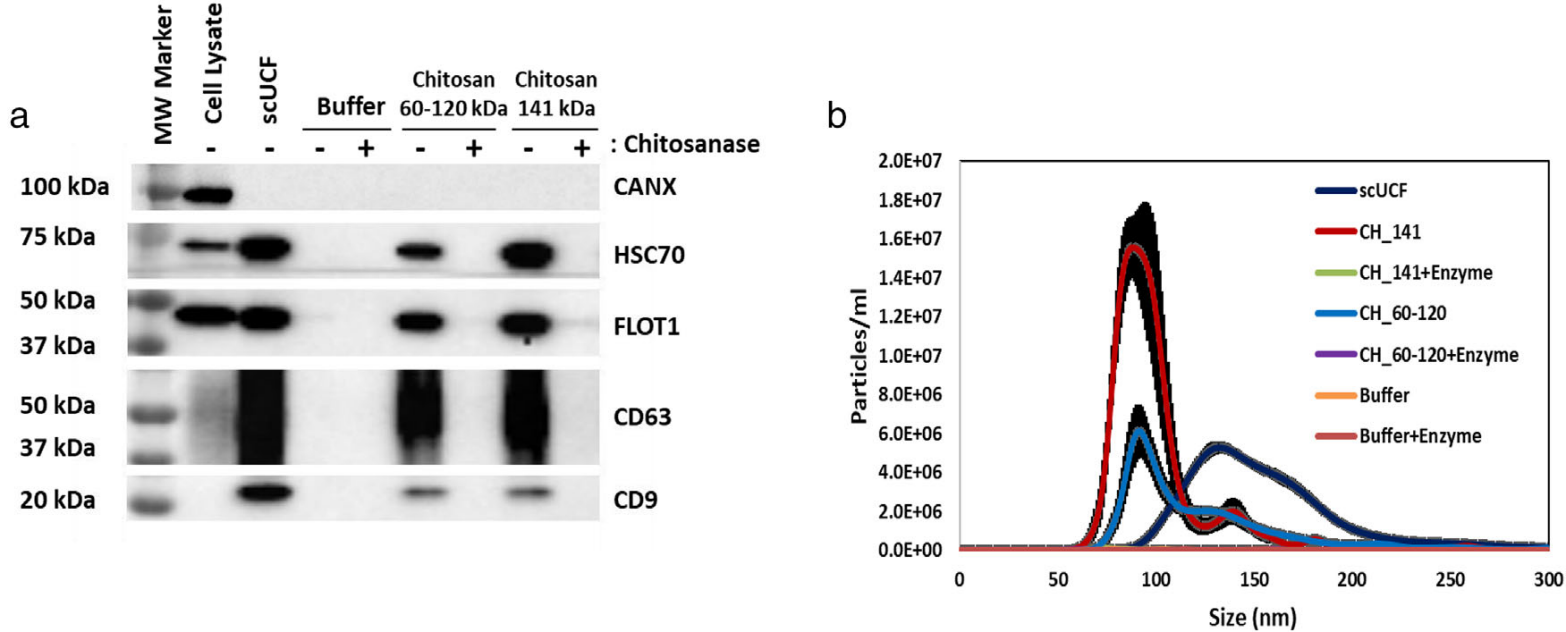
A polymeric form of chitosan is necessary to isolate sEVs from biological fluids. (a) Pre‐cleared HEK‐293 CCM (2 ml) was incubated with a final concentration of 50 μg/ml of the acidic formulation of chitosan that was either digested (+) or undigested (‐) with chitosanase (1U/mg of chitosan). Two chitosan types of different polymeric lengths, 60–120 kDa and 141 kDa, were used. Western blot analyses for CD63, CD9, HSC70, FLOT1, and CANX were performed on the isolated material; total cell lysate from MCF‐10A cells was used as a positive control for CANX. (b) NTA analysis of sEVs isolated from HEK‐293 CCM by scUCF or the acidic formulation of chitosan of either 60–120 kDa or 141 kDa, with (+enzyme) or without chitosanase treatment. Particle concentration (particles/ml) is plotted against size (nm) of particles. The mean plus the standard error of the mean (n = 3) is shown

We wished to further confirm that chitosan polymers are required for the isolation of sEVs, rather than small chitosan oligomers or the (1→4)‐2‐amino‐2‐deoxy‐β‐D‐glucan chitosan subunits. Towards this end, we used an enzyme known as chitosanase to digest chitosan into its oligomeric and/or monomeric glucan subunits (Izume et al., 1992). We also tested a different size chitosan polymer (141 kDa) to examine the specificity of polymer length for sEV isolation. 2 ml of CCM was incubated with chitosanase‐digested or intact, undigested chitosan, followed by low‐speed centrifugation to isolate sEVs. Isolation of sEVs by scUCF was included as a positive control, and addition of the acidic buffer (with or without chitosanase treatment) was included as a negative control. As expected, the intact, polymeric chitosan is able to isolate sEVs, as shown by the presence of canonical EV markers in a Western blot analysis of the isolated material (Figure 3a). In contrast, chitosanase‐digested chitosan does not isolate material that contains canonical EV markers (Figure 3a), suggesting that polymeric chitosan, and not oligomeric or monomeric chitosan subunits, is required for sEV isolation. Moreover, we found that 141 kDa chitosan is also able to isolate material with canonical EV markers (Figure 3a) indicating that larger chitosan polymers exhibit activity for sEV isolation.

In a parallel experiment we performed NTA to determine whether the different polymeric forms of chitosan can isolate similar sized sEVs and numbers. As described above, chitosan‐sEV complexes were incubated with 2 M NaCl solution to disperse individual sEVs prior to NTA. The size range of particles isolated with the 60–120 kDa chitosan is ∼75 nm to 170 nm, whereas the size range of particles isolated by scUCF is ∼90 nm to 200 nm (Figure 3b), as previously observed. We found that the 141 kDa chitosan isolates particles with a size range of ∼60 nm to 150 nm, similar to the size range for sEVs isolated by the 60–120 kDa chitosan (Figure 3b). One striking difference we observed is that the 141 kDa chitosan isolates more particles (5.31 × 10^8^ particles/ml), with an average size of ∼100 nm and mode size of ∼90 nm) than both 60–120 kDa chitosan (2.64 × 10^8^ particles/ml) and scUCF (4.08 × 10^8^ particles/ml). Moreover, the number of particles isolated by 141 kDa chitosan is greater than the number of particles isolated by either 60–120 kDa chitosan or scUCF (Figure 3b), suggesting that larger polymeric chitosan has higher activity for sEV isolation. The number of particles isolated by chitosanase‐digested chitosan of all polymeric sizes was negligible and therefore precluded a size analysis (Figure 3b). Altogether, these data indicate that polymeric chitosan is required for sEV isolation and that larger polymers may have higher activity for sEV isolation (Supplementary Table S1). Nonetheless, we continued to use 60–120 kDa chitosan to ensure a broad spectrum of polymeric sizes of chitosan were assayed in our experiments.

### 3.2 Chitosan isolates sEVs from human plasma

**FIGURE 4:**
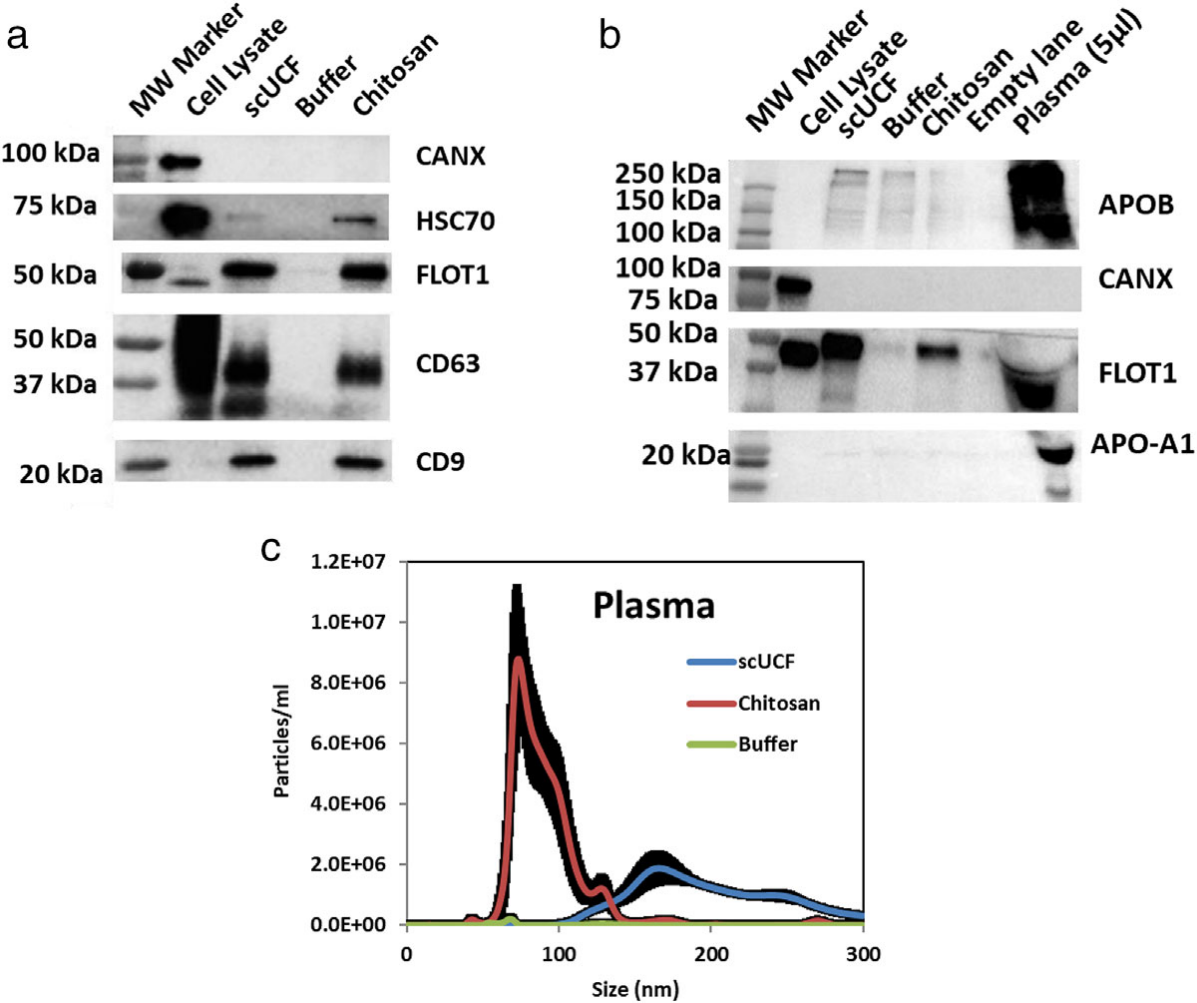
Chitosan‐mediated sEV isolation from human plasma. (a) Human plasma (0.25 ml) was diluted 1:4 with PBS and subjected to pre‐clearing as described in Figure 1, followed by sEV isolation by scUCF, the addition of the acidic formulation of chitosan to a final concentration of 50 μg/ml, or buffer. Western blot analyses of canonical EV markers CD63, CD9, HSC70, and FLOT1, as well as the non‐sEV marker CANX; total cell lysate from MCF‐10A cells was used as a positive control for CANX. (b) Western blot analyses of common contaminant plasma proteins APO‐A1 and APOB as well as FLOT1, a canonical EV marker; total cell lysate from HEK‐293 cells and 5 μl of plasma lysate (undiluted) were used as a positive controls for CANX, APO‐A1, and APOB. (c) NTA analysis of sEVs isolated from plasma by scUCF, the addition of the acidic formulation of chitosan to a final concentration of 50 μg/ml, or buffer. Particle concentration (particles/ml) is plotted against size (nm) of particles. Mean plus the standard error of the mean (n = 3) is shown

We next chose to determine if chitosan is able to isolate sEVs from a viscous fluid that is rich in proteins, such as plasma. Plasma from healthy volunteers was diluted four‐fold with PBS (0.25 ml of plasma supplemented with 0.75 ml of PBS) prior to preclearing at 17,000 × *g* for 15 min. The diluted and precleared plasma was incubated with a final concentration of 50 μg/ml chitosan; the addition of acidic buffer or scUCF was included as negative and positive controls, respectively. The isolated material was probed for canonical EV protein markers by Western blot. We observed appreciable amounts of CD63, CD9, HSC70, and FLOT1 in the material isolated with chitosan and the scUCF‐isolated material (Figure 4a), whereas material isolated by the addition of the acidic buffer did not display any signal for the canonical EV markers. The absence of a signal for CANX was observed in both the chitosan‐ and scUCF‐isolated material (Figure 4a) indicating the material isolated by these methods is devoid of cellular contamination. Moreover, we also probed for the presence of APO‐A1 and APOB in our isolated materials, two high‐abundance plasma proteins that are common contaminants of EV isolation methods due to their ability to form particles that are similar in size and density to EVs (Yuana et al., 2014). We found that chitosan‐ and scUCF‐isolated material contains little, if any, signal for these high‐abundance plasma proteins (Figure 4b).Next, we performed Western blot analyses to detect human lectins that are present in plasma at high abundance (Beulaja Manikandan et al., 2020) and may co‐isolate in EV preparations, including H‐Ficolin (FCN3), tetranectin (TETN), and serum amyloid A (SAA (Supplemental Figure S3). Protein lysates from plasma collected pre‐ and post‐chitosan incubation were included as controls. Our analysis showed that the lectins are either absent or present in very low abundance in chitosan‐isolated material, indicating that chitosan does not co‐isolate abundant non‐EV proteins from plasma (Supplemental Figure 3). These findings indicate that chitosan is able to isolate material from human plasma that is enriched for canonical EV marker proteins.

We next sought to analyse particle concentration and size distribution of particles isolated from plasma using chitosan by NTA. 2 ml of diluted plasma was incubated with a final concentration of 50 μg/ml chitosan to isolate material; scUCF and the acidic buffer were also performed as positive and negative controls, respectively. We observed a size distribution of ∼60 nm to 140 nm for chitosan‐isolated material, whereas scUCF‐isolated material has a size distribution of ∼110 nm to 300 nm (Figure 4c), indicating this method isolates larger particles or EVs from plasma. The concentration of particles isolated with chitosan (2.94 × 10^8^ particles/ml) was higher than the concentration of particles isolated by scUCF (2.18 × 10^8^ particles/ml) (Figure 4c). The acidic buffer isolated negligible amounts of material and therefore we were unable to determine the size range. These results indicate that chitosan isolates particles within the appropriate size range for sEVs, providing further evidence that chitosan is able to isolate sEVs from plasma (Supplementary Table S1).

We also examined the morphology of the particles isolated from plasma by chitosan using TEM. The material isolated from plasma by both chitosan and scUCF showed membrane‐bound vesicle structures; however, no membrane‐bound structures were observed for the material isolated by the addition of the acidic buffer (Supplemental Figure S4). These results provide additional evidence that chitosan mediates the isolation of sEVs from plasma.

### 3.3 Chitosan isolates sEVs from a variety of body fluids

**FIGURE 5:**
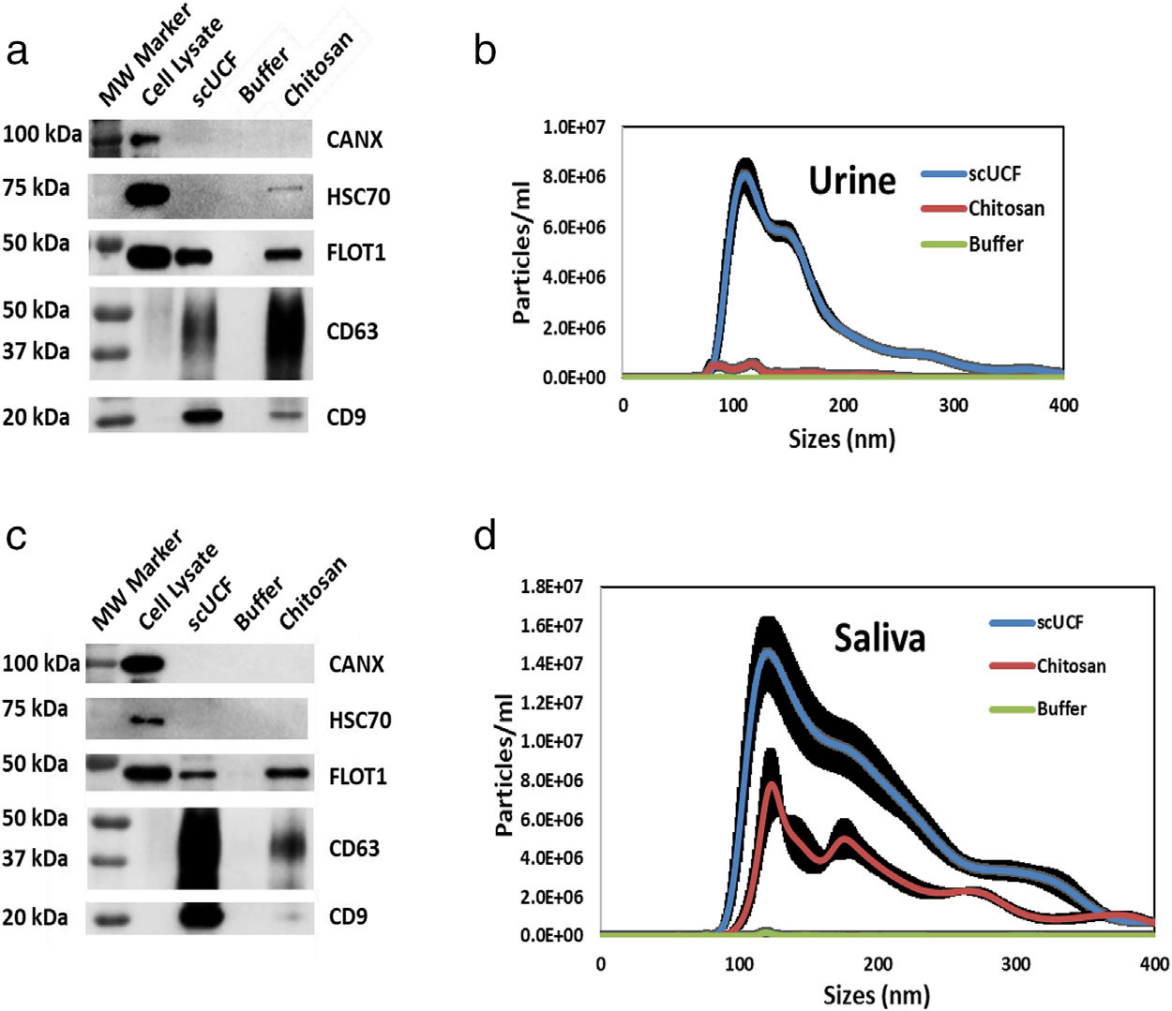
Chitosan‐mediated sEV isolation from human urine and saliva. sEVs were isolated from (a) 1 ml of urine or (c) 1 ml of saliva by scUCF, the addition of the acidic formulation of chitosan (60‐120 kDa) to a final concentration of 50 μg/ml, or buffer followed by Western blot analyses of canonical EV markers CD63, CD9, HSC70, and FLOT1. Blots were also probed for CANX, a non‐sEV marker; total cell lysate from MCF‐10A cells was used as a positive control for CANX. NTA analysis of sEVs isolated from (b) urine or (d) saliva by scUCF, a final concentration of 50 μg/ml of the acidic formulation of chitosan, or buffer. Particle concentration (particles/ml) is plotted against size (nm) of particles. Mean plus the standard error of the mean (n = 3) is shown

To determine whether chitosan can isolate sEVs from a wide variety of human biofluids, we next assayed chitosan for the isolation of sEVs from urine and saliva. Urine was collected from healthy volunteers and subjected to pre‐clearing followed by the addition of chitosan to 1 ml of urine to a final concentration of 50 μg/ml; scUCF and acidic buffer were included as controls. Western blot analyses of the isolated material showed the presence of the canonical EV markers CD63, CD9, HSC70, and FLOT, as well as the absence of the non‐sEV protein CANX, for both the chitosan‐ and scUCF‐isolated materials (Figure 5a). The acidic buffer was unable to isolate material that contains any of the canonical EV markers. These results show that chitosan is able to isolate material from urine that contains canonical EV marker proteins. Western blot analysis for Tamm‐Horsfall protein (THP), an abundant protein present in urine (Fernández‐Llama et al., 2010), was performed to assess the co‐isolation of non‐EV proteins from urine by chitosan. We find that both chitosan and scUCF co‐isolate THP (Supplemental Figure S5), indicating that chitosan does isolate some non‐EV material from urine, although at a level similar to that obtained by scUCF.

To determine the size distribution of material isolated from urine by chitosan we performed NTA. Once again, 1 ml of urine was subjected to the addition of chitosan to a final concentration of 50 μg/ml, scUCF, or the addition of acidic buffer. Material isolated by chitosan was found to have a size range of ∼80 nm to 140 nm, whereas the size range of material isolated by scUCF has a size range of ∼80 nm to 300 nm (Figure 5b). The concentration of particles for chitosan‐mediated isolation (4.17 × 10^7^ particles/ml), although significant, was found to be lower than the concentration of particles isolated by scUCF (7.40 × 10^8^ particles/ml) (Figure 5b). The acidic buffer isolated negligible numbers of particles. These data suggest that chitosan isolates particles within the size range of sEVs, albeit a lower particles count was observed compared to scUCF (Supplementary Table S1).

Next, we examined the morphology of the particles isolated from urine by chitosan. The material isolated from urine with chitosan showed membrane‐bound vesicle structures, similar to structures observed in scUCF‐isolated material from the same urine sample (Supplemental Figure S6). No membrane‐bound structures were observed for the material isolated by the addition of the acidic buffer (Supplemental Figure S6). The presence of dark patches in the background of the TEM images for material isolated from urine may be due to urochrome precipitation or any non‐specific sedimentation (Fogazzi & Garigali, 2003; Wald et al., 2009). These results indicate that chitosan isolates membrane‐bound structures with the morphological characteristics of sEVs from human urine.

We further sought to test ability of chitosan to isolate sEVs from human saliva. Commercially‐available saliva was precleared of cells, cell debris, apoptotic bodies and large vesicles by a two‐step centrifugation at 3,000 × *g* and 17,000 × *g*. Chitosan was added to a final concentration of 50 μg/ml to 1 ml of precleared saliva; scUCF and acidic buffer were included as positive and negative controls, respectively. We were able to detect the canonical EV marker proteins FLOT1, CD63, and CD9 by Western blot analyses of both the chitosan‐ and scUCF‐isolated material (Figure 5c); however, HSC70 was not detected in any of the material isolated by either chitosan or scUCF. No signals for any canonical EV marker were detected in material isolated by the addition of the acidic buffer, and a signal for the non‐sEV protein CANX was not detected in either chitosan‐ or scUCF‐isolated material (Figure 5c). These results show that chitosan is able to isolate material with canonical EV marker proteins from human saliva.

We analysed the size distribution of material isolated by chitosan from the same batch of saliva by performing NTA. Chitosan was added to 1 ml of precleared saliva to a final concentration of 50 μg/ml; scUCF and acidic buffer were included as controls. Particles isolated with chitosan have a size range of ∼90 nm to 350 nm and a concentration of 8.39 × 10^8^ particles/ml, whereas particles isolated by scUCF have a size range of ∼90 nm to 350 nm and a concentration of 1.89 × 10^9^ particles/ml (Figure 5d). The addition of acidic buffer to saliva failed to isolate an appreciable number of particles for NTA analysis. These results demonstrate that chitosan is able to isolate particles within the size range of sEVs from human saliva (Supplementary Table S1).

We examined the morphology of the particles isolated from saliva by chitosan using TEM. The material isolated from saliva by both chitosan and scUCF showed membrane‐bound vesicle structures; however, no EV‐like structures were observed in the material isolated by the addition of the acidic buffer alone (Supplemental Figure S7). These findings provide additional support for chitosan's ability to isolate sEVs from human saliva.

Taken together, our data indicate that chitosan can isolate sEVs from diverse biofluids, each with a different physicochemical complexity, suggesting that chitosan activity for sEV isolation is compatible with many types of biological fluid.

### 3.4 Proteomic analyses of chitosan‐isolated sEVs from CCM, urine and saliva

We next chose to analyse the proteome of the material isolated from CCM, urine and saliva with chitosan to determine whether this material contains proteins that are representative of sEVs and/or exosomes by performing mass spectrometry (MS) followed by Gene Ontology analysis, a further confirmation that chitosan can isolate sEVs from these biological fluids. For these experiments we included a proteome analysis of sEVs isolated by scUCF as a positive control. The total protein from the isolated material was resolved using 10% SDS‐PAGE and visualized using EZblue; the visualized lane was subdivided into 12 sections, which were excised and subjected to proteomic analysis by MS. Protein samples from each isolation condition were analysed in triplicate and proteome lists were prepared with proteins that showed a minimum of two representative peptides as a “cut off” and that were identified in at least two out of three replicates. The number of common proteins among all three replicates for each method (scUCF and chitosan) and for all three biological sample types (CCM, urine and saliva) is shown in Supplemental Figure S8 to demonstrate the commonality of proteins identified in experimental replicates. The complete list of identified proteins and similarities between the chitosan and scUCF isolation methods for each biofluid is provided in the Supplementary Excel file S1.

**FIGURE 6:**
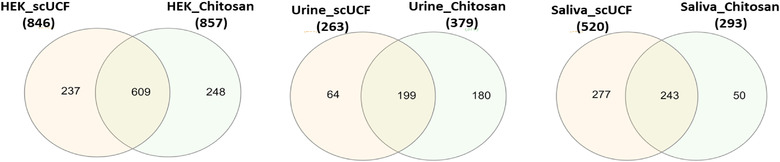
Venn diagrams showing common sEV proteins isolated by scUCF and the acidic formulation of chitosan from (a) HEK‐293 CCM, (b) urine, and (c) saliva. All the samples were run in triplicate for mass spectrometry analysis and the proteins considered for comparative analysis and gene ontology were identified in at least two out of three technical replicates

For experiments that used CCM as a source of sEVs, the total number of proteins identified from material isolated by scUCF and chitosan were 846 and 857, respectively (Figure 6a). A total of 609 proteins from material isolated by chitosan were found in common with the proteins identified in the sEVs isolated by scUCF, representing 71% of the proteins identified in the chitosan‐isolated material (Figure 6a). These results indicate that the majority of proteins found in the material isolated by the addition of chitosan to CCM are also found in sEVs isolated by scUCF. For experiments that used urine as a source of sEVs, the total number of proteins identified from material isolated by scUCF and chitosan were 263 and 379, respectively (Figure 6b). The chitosan‐isolated material had 199 proteins in common with scUCF‐isolated sEVs, representing 53% of the proteins identified in the chitosan‐isolated material (Figure 6b). These results indicate that the majority of proteins found in the material isolated by the addition of chitosan to urine are also found in sEVs isolated by scUCF. When saliva was used as a source of sEVs, the total number of proteins identified from material isolated by scUCF and chitosan were 520 and 293, respectively (Figure 6c). The chitosan‐isolated material has 243 proteins in common with scUCF‐isolated sEVs, representing 83% of the proteins identified in the chitosan‐isolated material (Figure 6c). These results show that the majority of proteins found in the material isolated by the addition of chitosan to saliva are also found in sEVs isolated by scUCF. Altogether, these data show that the majority of proteins identified in chitosan‐isolated material from CCM, urine, and saliva are also found in sEVs isolated from the same biofluids using scUCF.

In order to determine that the proteins identified in chitosan‐isolated material and scUCF‐isolated sEVs are *bona fide* EV‐associated proteins, we analysed the above data in silico by Gene Ontology (GO) analysis for subcellular components. As shown in Table 1, the GO analyses for ‘exosome’ (in cellular component clusters) were correlated using two different online programs (FunRich and PantherDB). We chose two GO analysis programs to observe any differences in cellular component clustering analysis that could arise from different background annotations used to generate GO terms in these two programs. The corrected p‐values produced by each analysis are highly significant and comparable for both FunRich and Panther software, indicating the proteome datasets for our chitosan‐ and scUCF‐isolated material are a subset of the proteins identified by the GO term ‘exosome’. The percentage matches were in the range of 57%–76% in case of material isolated from CCM, whereas in case of urine and saliva the percentage matches were greater than 80% of proteins identified by the GO term ‘exosome’ (Table 1). Moreover, the FunRich analysis of the proteins identified from chitosan‐ and scUCF‐isolated material revealed a 5–6 fold enrichment for ‘exosome’ as a subcellular component; however, Panther analysis revealed a 6–9 fold enrichment (Table 1). The fold enrichment shows over‐representation compared to the background and is calculated by dividing the observed values by expected outcomes. The outputs of both FunRich and Panther gene ontology software were comparable in this study, thereby providing an independent assessment that proteins from chitosan‐isolated sEVs are mostly categorized as ‘exosome’ and are comparable to proteins from scUCF‐isolated sEVs.

**TABLE 1:** Proteomics‐based‐GO analysis for exosomes as cellular components

	FUNRICH			PANTHER		
	Percentage of genes	Fold enrichment	Corrected *P*‐value (Bonferroni method)	Percentage of genes	Fold enrichment	FDR corrected *P*‐value
HEK ‐scUCF	68.63	4.94	1.9E‐304	61.73	6.18	2.08E‐281
HEK‐Chitosan	64.02	4.61	4.3E‐264	57.92	5.80	3.28E‐248
Urine ‐scUCF	91.78	6.60	4.4E‐176	93.43	9.36	8.06E‐221
Urine‐Chitosan	85.21	6.13	2.1E‐212	92.21	9.24	2.52E‐308
Saliva ‐scUCF	84.24	6.06	1.5E‐273	80.96	8.11	1.20E‐311
Saliva‐Chitosan	86.87	6.25	1.1E‐155	83.50	8.36	1.15E‐189

We also performed a comparative analysis of our proteomic data for chitosan‐isolated material using the human protein data previously reported in Vesiclepedia (Pathan et al., 2019) (Supplementary Figure S9 and Supplementary Excel file S2). We found that ∼94% of the proteins identified in chitosan‐isolated material from HEK‐CCM, ∼92% of proteins identified from urine, and ∼89% of proteins identified from saliva were previously found in human EVs (cell lines or biofluids) and reported in Vesiclepedia. These results further support the conclusion that chitosan is capable of isolating sEVs from these biofluids.

To expand on our findings, we performed a mass spectrometry analysis in which we compared the proteome of chitosan‐isolated material to the proteome of EVs that were isolated using sucrose density gradient ultracentrifugation, a method that separates EVs based on density (Taylor & Shah, 2015). We identified the EV‐enriched fractions of the sucrose density gradient by performing Western blot analyses for the EV markers CD63 and CD9 (Supplemental Figure S10). The EV‐enriched fractions (F2, F3, and F4) were pooled and processed for mass spectrometry analysis, along with chitosan‐isolated material, as described above. A total of 423 of 1257 proteins identified in chitosan‐isolated EVs were also found in density gradient‐isolated EVs (Supplementary Figure S11, Supplementary Excel file S3). The proteins lists were then analysed by FunRich for cellular component (Supplementary Figure S11). More than 40% of the 834 proteins identified in chitosan‐isolated material, but not in density gradient‐isolated EVs, are assigned to “Exosomes” with 2.9‐fold enrichment (Supplementary Figure S11, Supplemental Table S2), suggesting that despite not being identified in the EV‐enriched gradient fractions these proteins are *bona fide* EV proteins. These findings provide further support for the conclusion that chitosan facilitates the isolation of sEVs.

### 3.5 Chitosan attached to magnetic beads facilitates sEV isolation

**FIGURE 7:**
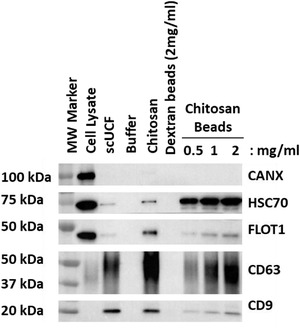
sEV isolation by chitosan‐coated magnetic beads. Increasing amounts of chitosan‐coated magnetic beads (0.5, 1, and 2 mg/ml) were added to 1 ml of HEK‐293 CCM; dextran‐coated magnetic beads (2 mg/ml) or buffer served as a negative controls and scUCF or the addition of the acidic formulation of chitosan to a final concentration of 50 μg/ml were used as positive controls. Western blot analyses of canonical EV markers CD63, CD9, HSC70, and FLOT1, as well as the non‐sEV marker CANX were performed for material isolated from HEK‐293 CCM; total cell lysate from HEK‐293 cells was used as a positive control for CANX

Our results demonstrate that the addition of chitosan to CCM, as well as to a wide variety of biological fluids, is able to facilitate the isolation of sEVs by low‐speed centrifugation. We next chose to test the platform versatility of chitosan by determining if chitosan that is immobilized on a magnetic bead is also capable of mediating sEV isolation from CCM. Towards this end, magnetic beads coated with chitosan were added to 1 ml of CCM at increasing concentrations (ranging from 0.5 mg/ml to 2 mg/ml); a final concentration of 2 mg/ml of dextran magnetic beads, an unrelated polysaccharide, were used as a negative control to identify any non‐specific binding of sEVs to beads coated with a polysaccharide other than chitosan. We also included the addition of chitosan to a final concentration of 50 μg/ml and scUCF as positive controls for sEV isolation. Chitosan‐coated magnetic beads at a final concentration of 0.5 mg/ml were found to capture material that gives a positive signal for the canonical EV markers HSC70, CD63, CD9, and FLOT1, but not the non‐sEV marker CANX, by Western‐blot analyses (Figure 7). A dose‐dependent increase in signal for the canonical EV markers was observed with increasing concentrations of chitosan‐coated magnetic beads, up to a final concentration of 2 mg/ml (Figure 7). Material that was isolated by the dextran magnetic beads at a final concentration of 2 mg/ml did not have any signal for the canonical EV markers by Western blot (Figure 7), indicating that sEVs are specifically bound to chitosan‐coated beads rather than polysaccharide‐coated beads in general. These results show that chitosan that has been immobilized on magnetic beads is capable of isolating sEVs, demonstrating the versatility and adaptability of chitosan to various platforms for sEV isolation.

## 4 DISCUSSION

Recently small EVs, especially exosomes, have received attention from researchers, clinicians and entrepreneurs because of their therapeutic and diagnostic potential for many diseases. Here we report a simple, robust, and versatile low‐speed sEV isolation method that uses the polysaccharide chitosan, a biocompatible, biodegradable, non‐toxic, and non‐immunogenic molecule (Cheung et al., 2015) that has been FDA‐ and EU‐approved for wound dressing (Wedmore et al., 2006) and dietary applications (Huang et al., 2020).

The currently‐used standard method for sEV isolation is differential centrifugation followed by ultracentrifugation (UCF; either sucrose cushion or density gradient) (Patel et al., 2019), which is practised primarily in research laboratories. Commercially‐available methods that have been developed to reduce the time required for EV isolation and/or reduce the need for specialized equipment (e.g. an ultracentrifuge) include polymer‐assisted (PEG or its derivatives) precipitation (Momen‐Heravi et al., 2015), immuno‐affinity purification (Greening et al., 2015), size‐exclusion chromatography (SEC) (Böing et al., 2014) and microfluidics (Santana et al., 2014). Each of these methods has challenges for clinical compatibility (Konoshenko et al., 2018). UCF is not only infrastructure‐reliant but also co‐sediments large, non‐specific protein aggregates and high‐density lipoproteins (HDLs) (Yuana et al., 2014). Moreover, reports have shown that UCF physically damages EVs (Linares et al., 2015; Nordin et al., 2015), which may affect functionality and thereby render UCF‐isolated EVs unsuitable for therapeutic applications (Van Deun et al., 2014). Contamination and retention of the respective polymers are major toxicity concerns for polymer‐based methods (Konoshenko et al., 2018). Antibody‐based methods yield relatively pure EVs, but are associated with batch‐to‐batch variation of antibodies, difficulties with detachment of molecules, and nonspecific binding (Greening et al., 2015). SEC usually co‐elutes large protein aggregates and lipoproteins along with EVs (Böing et al., 2014; Konoshenko et al., 2018).

Chitosan is a commercially‐available polysaccharide and is produced by deacetylation of chitin (Elieh‐Ali‐Komi & Hamblin, 2016), which originates from the cell walls of different fungi and the exoskeletons of crustaceans. Differences in molecular weight and the percentage of deacetylation determine the physicochemical properties of specific chitosan species for different biomedical (Elieh‐Ali‐Komi & Hamblin, 2016) and cosmeceutical (Aranaz et al., 2018) applications. Chitin is composed of two monomer units present in different fractions: (i) 2‐acetamino‐2‐deoxy‐d‐glucopyronase (N‐acetyl‐d‐glucosamine) and (ii) 2‐amino‐2‐deoxy‐d‐glucopyronase (N‐amino‐d‐glucosamine). The first unit, N‐acetyl‐d‐glucosamine, is mainly responsible for the insolubility of chitin as strong hydrogen bonds occur between the acetyl groups of the same or adjacent chitin chains. During the process of deacetylation, when treated with alkaline solution, the amino sites of N‐amino‐d‐glucosamine react with aldehyde and ketone groups resulting in Schiff Bases formation and influence solubility. Chitosan is soluble at low pH (pH < 6) due to the protonation of its primary amino groups. At high pH (pH > 6.0) the primary amino groups are deprotonated and hydrogen bonds involving neutralized NH_2_ groups induce agglomeration and reduce chitosan's ability to associate with other molecules *via* electrostatic interactions (Bellich et al., 2016). Like most biological membranes, sEVs also have a net negative zeta potential (Vogel et al., 2017), common for any nano‐ or nano‐bio dispersive systems (Xu et al., 2012). The zeta potential of sEVs varies from approximately ‐10 mV to ‐20 mV (Helwa et al., 2017) and acquires different surface electrical charges based on the ionic strength of the media, biofluids, or storage media, such as urine and PBS, respectively, which are polar and hydrophilic (Beit‐Yannai et al., 2018). These factors may explain why the acidic formulation of chitosan performs better than the neutral formulation for sEV isolation (Figure 2). Other factors may also influence this interaction, for example nanoparticle‐polymer‐crowding based phase‐separation of soft matter, mainly predominant in biological systems (Denton, 2014). Higher viscosity is one of the salient properties of different biofluids due to presence of proteins like albumin. For example, plasma, urine and saliva have protein concentration averages of 70.0, 0.1, 1.0 mg/ml, respectively (Parviainen et al., 2011; Shaila et al., 2013; Yang et al., 2015). Another example of extreme variation of constituents is urea content in body fluids (for example, plasma, urine and saliva; average urea content: 9 mM, 700 mM and 7.5 mM respectively) (Lasisi et al., 2016; Saatkamp et al., 2016). These variable physical properties may explain why most commercial EV‐isolation kits do not exhibit uniform efficiency when applied to different body fluids. We believe wide variations in physicochemical properties of different biofluids also influence the efficacy of sEV isolation using acidic and neutral chitosan formulations. As observed in case of urine and saliva, the capture and/or elution efficiency for chitosan‐mediated sEV isolation is variable and dependent on the biofluid source of sEVs (Figure 5). This may be due to variability in the physical properties (e.g. viscosity and density) or chemical properties (e.g. pH and ionic strength) of the biofluids, which may ultimately result in less efficient capture or elution of sEVs from chitosan‐EV complexes for both urine and saliva.

sEVs carry cargo capable of intercellular communication by delivering information to other cells in the form of proteins, nucleic acids, lipid and metabolites (van Niel et al., 2018). To better understand that composition of sEVs isolated with chitosan we elucidated proteomic profiles by mass spectroscopy to generate a list of protein cargo present in the isolated sEVs. In parallel we performed a comprehensive proteomic analysis of sEVs isolated by the scUCF method as our standard for comparison. The resulting protein lists were analysed for “exosome” (cellular component) using two gene ontology (GO) tools: FunRich (Pathan et al., 2015) and Panther (Protein ANalysis THrough Evolutionary Relationships) (Mi et al., 2013) to determine if the proteome of chitosan‐isolated sEVs is highly similar to sEVs in general. Both tools produced highly significant and similar GO‐confidence (p‐values) for cellular component ‘exosome’ for the chitosan‐isolated sEVs when compared with the UCF‐isolated sEVs from the same samples (Table 1). UCF sEV isolation is the “gold standard” and is believed to yield a fairly pure sEV preparation (Greening et al., 2015). Western blot analyses that compared the chitosan‐isolated sEV and scUCF‐isolated sEV protein cargoes produced differential signal intensity for the sEV protein markers examined (Figures 2, 4 and 5). We noted that CD9 abundance in chitosan‐isolated sEV preparations from different biofluids, including CCM, urine, and saliva, was lower than that observed in sEVs isolated by scUCF and that the size distribution profile of sEVs captured by chitosan from CCM and plasma were markedly different than that of scUCF sEVs. Moreover, we also observed for our proteomic data (Figure 6) that although a large subset of proteins is common between chitosan‐isolated sEVs and scUCF‐isolated sEVs, these protein lists are not identical. These results raise the possibility that chitosan may isolate a subset of sEVs and/or co‐isolates other types of nanoparticles along with sEVs. Our comparative proteomic analysis of density gradient‐isolated material and chitosan‐isolated material from the conditioned culture media of HEK 293 cells provides additional support for this possibility since several proteins are identified in the chitosan‐isolated material but not in the density‐gradient isolated material (Supplementary Figure S11). Nonetheless, our results clearly show that chitosan captures a population of sEVs, but also suggest that chitosan may have an affinity for other nanoparticles or extracellular protein complexes that are present in conditioned culture media. These findings are similar to those observed for EV isolation with other polysaccharide heparin, which was found to co‐isolate a set of proteins not previously found to be associated with EVs (including centrosomal proteins, which we also identified for chitosan‐isolated material) (Balaj et al., 2015). Also, this is consistent with other reports that show that the characteristics of isolated EVs, including their size and cargo, can vary depending on the biophysical method used for their isolation (Nath Neerukonda et al., 2019; Neerukonda et al., 2020). EVs are considered promising vehicles for therapeutic applications because of their ability to penetrate and deliver therapeutic cargo across cell membranes and the blood‐brain barrier (Shahjin et al., 2020). Furthermore, EVs harvested from mesenchymal stem cells have been demonstrated to be powerful agents for cell‐free therapy (Zhang et al., 2019), heightening future potential for EV‐based therapeutic applications. Tremendous effort has been applied in recent years to develop and optimize new technologies to achieve the therapeutic potential of EVs (Yamamoto et al., 2019). The first major challenge is to develop and refine large‐scale EV isolation methods that are compatible with therapeutic applications. Most established EV isolation methods have drawbacks that preclude their use for the isolation of therapeutic EVs, including high cost, scalability, purity, integrity, and dilution (Yamashita et al., 2018).

Chitosan's wide range of proven clinical and preclinical research applications (Mohammed et al., 2017), suggests that the presence of residual chitosan in sEV preparations may not limit the therapeutic application of the sEVs isolated by this method, since chitosan is non‐toxic. Moreover, chitosan‐mediated sEVs isolation uses low‐speed centrifugation, which may minimize physical damage to the sEVs that could affect their functionality. Therefore, chitosan‐mediated sEVs isolation yields physically intact sEVs that likely have low toxicity, and along with the low cost of chitosan and its potential scalability, raises the possibility of their use in therapeutic applications. Future studies will assess the use of chitosan‐isolated sEVs for therapeutic delivery. Decades of chitosan's topical (wound healing) and dietary uses have demonstrated its safety for therapeutic applications, and therefore place this method of EV isolation in an advantageous position for future therapeutic usage.

## CONFLICT OF INTEREST

All the authors report that there is no conflict of interest.

## AUTHOR CONTRIBUTIONS

Conceived the study: Anirban Ghosh; Experimental planning and design: Awanit Kumar and Anirban Ghosh; Performed experiments, and analysed data: Awanit Kumar, Surendar Reddy Dhadi and Anirban Ghosh; Wrote, organized and edited the manuscript: Awanit Kumar, Surendar Reddy Dhadi, Anirban Ghosh, Catherine Taylor, Jeremy W. Roy, Stephen M. Lewis and Rodney J. Ouellette; Mass spectrometry analysis: Ngoc‐Nu Mai and David A. Barnett; Funding acquisition and overall supervision: Anirban Ghosh, Stephen M. Lewis and Rodney J. Ouellette. All the authors have read the manuscript and approved for publication.

## Supporting information

Supporting Information

Supporting Information

Supporting Information

Supporting Information
