# Supplementary material for: The polysaccharide chitosan facilitates the isolation of small extracellular vesicles from multiple biofluids
Source: J Extracell Vesicles. 2021 Sep 1;10(11):e12138. doi: 10.1002/jev2.12138 (PMC8409086; doi:10.1002/jev2.12138)

**Supplementary Table 1: NTA data with normalized particle counts per mL of starting biofluids**

| **Sample** | **Particles/mL** | **SE^*^** | **Mode (nm) +/- SE^*^** | **Mean (nm) +/- SE^*^** |
| --- | --- | --- | --- | --- |
| HEK_scUCF | 4.08E+08 | 9E+06 | 133.2 +/- 2.6 | 164.8 +/- 1.6 |
| HEK_Chitosan-Acidic | 2.02E+08 | 1.14E+07 | 125.3 +/- 1.7 | 133.0 +/- 5.2 |
| HEK_Acidic Buffer | 0 | 0.00E+00 | 0 | 0 |
| HEK_Chitosan-Neutral | 3.47E+07 | 5.17E+06 | 127.3 +/- 12.6 | 158.8 +/- 9.2 |
| HEK_Neutral Buffer | 9.96E+05 | 5.00E+05 | 65.6 +/- 30.9 | 65.3 +/- 31.0 |
| **Without and with Chitosanase (Enzyme) treated samples** | | | | |
| HEK_CH_60-120 | 2.64E+08 | 2.37E+07 | 99.8 +/- 7.1 | 131.2 +/- 5.2 |
| HEK_CH_141 | 5.31E+08 | 6.88E+07 | 88.7 +/- 2.4 | 100.3 +/- 1.3 |
| HEK_Buffer | 1.73E+06 | 766095.2 | 129.0 +/- 30.7 | 125.8 +/- 30.6 |
| HEK_CH_60-120+Enzyme | 1.64E+06 | 6.44E+05 | 86.2 +/- 20.4 | 86.0 +/- 15.1 |
| HEK_CH_141+Enzyme | 2.34E+06 | 8.51E+05 | 79.3 +/- 6.2 | 76.6 +/- 6.9 |
| HEK_Buffer+Enzyme | 1.50E+06 | 357172.9 | 117.5 +/- 18.2 | 209.3 +/- 49.9 |
| **Plasma** | | | | |
| Plasma_scUCF | 2.18E+08 | 3.24E+07 | 175.6 +7.1 | 222.1 +/- 6.1 |
| Plasma_Chitosan | 2.94E+08 | 4.24E+07 | 75.7 +/- 2.5 | 89.8 +/- 2.5 |
| Plasma_Buffer | 3.54E+06 | 2.29E+06 | 39.2 +/- 25.8 | 39.0 +/- 26.1 |
| **Urine** | | | | |
| Urine_scUCF | 7.40E+08 | 2.78E+07 | 110.2 +/- 1.5 | 165.2 +/- 0.8 |
| Urine_Chitosan | 4.17E+07 | 1.34E+06 | 112.7 +/- 13.3 | 136.4 +/- 5.1 |
| Urine_Buffer | 1.10E+06 | 4.89E+05 | 78.6 +/- 38.7 | 78.6 +/- 38.9 |
| **Saliva** | | | | |
| Saliva_scUCF | 1.89E+09 | 2.47E+08 | 120.7 +/- 1.0 | 205.7 +/- 2.3 |
| Saliva_Chitosan | 8.39E+08 | 4.06E+07 | 128.8 +/- 4.1 | 218.2 +/- 5.4 |
| Saliva_Buffer | 3.82E+06 | 7.13E+05 | 117.6 +/- 13.8 | 153.8 +/- 19.1 |

*SE: Standard Error

**Supplementary Table 2: FunRich-based GO Analysis of EVs proteins from HEK-CCM for “Exosomes” as cellular components**

|  | **Percentage of genes** | **Fold Enrichment** | **Corrected p-value  (Bonferroni method)** |
| --- | --- | --- | --- |
| Common_DG and Chitosan | 81.49 | 5.80 | 4.2E-217 |
| Only_Chitosan | 40.74 | 2.90 | 8.89E-78 |
| Only_DG | 55.67 | 3.97 | 5.27E-19 |

**SUPPLEMENTARY FIGURE LEGENDS**

**Supl. Figure 1**: Optimization of chitosan-mediated sEV isolation from HEK-293 CCM. Western blot analyses of canonical EV markers CD63, CD9, HSC70, and FLOT1 for material isolated by the addition of increasing concentrations (10-200 µg/mL) of the acidic or neutral formulations of chitosan (60-120 kDa) to 2 mL of CCM. scUCF-isolated sEVs from 2 mL of same CCM were used as a positive control. The acidic or neutral buffer in which chitosan was dissolved were included as negative controls.

**Supl. Figure 2**: TEM images of sEVs isolated by scUCF, chitosan (acidic formulation), and buffer from HEK-293 CCM. Scale bars represent 500nm or 200nm, as indicated. Membrane-bound structures of sEVs are indicated by white arrows.

**Supl. Figure 3**: Evaluation of human lectin co-isolated with chitosan-isolated sEVs from human plasma. Human plasma (0.25 mL) was diluted 1:4 with PBS and subjected to pre-clearing as described in Figure 1, followed by sEV isolation by scUCF, the addition of the acidic formulation of chitosan (60-120 kDa) to a final concentration of 50 µg/mL, or buffer. Western-blot analyses were performed for H-Ficolin (FCN3, 58 kDa), Tetranectin (TNA, 20 kDa), Serum amyloid A (SAA, 12 kDa) and FLOT1 (a canonical EV marker). Total cell lysate from HEK-293 cells and 5 µL of plasma (undiluted) were included as controls. For analysis of material in the plasma post-sEV isolation, 20 µL of supernatant from buffer (Supernatant_Buffer) and chitosan (Supernatant_Chitosan) isolations were included.

**Supl. Figure 4**: TEM images of sEVs isolated by scUCF, chitosan (acidic formulation), and buffer from Plasma. Scale bars represent 500nm or 200nm, as indicated. Membrane-bound structures of sEVs are indicated by white arrows.

**Supl. Figure 5**: Evaluation of THP (uromodulin) co-isolation with chitosan-isolated sEVs from human urine. sEVs were isolated from 1 mL of urine by scUCF, the addition of the acidic formulation of chitosan (60-120 kDa) to a final concentration of 50 µg/mL, or buffer followed by Western blot analyses of THP (uromodulin).

**Supl. Figure 6**: TEM images of sEVs isolated by scUCF, chitosan (acidic formulation), and buffer from urine. Scale bars represent 500nm or 200nm, as indicated. Membrane-bound structures of sEVs are indicated by white arrows.

**Supl. Figure 7**: TEM images of sEVs isolated by scUCF, chitosan (acidic formulation), and buffer from saliva. Scale bars represent 500nm or 200nm, as indicated. Membrane-bound structures of sEVs are indicated by white arrows.

**Supl. Figure 8:** Venn diagrams to demonstrate the commonality of sEV proteins among triplicate experiments for mass spectrometry analyses of (a) HEK-293 CCM, (b) urine, and (c) saliva. Using this analysis, a list of proteins identified in at least two out of three replicates was generated for the scUCF and chitosan-isolated sEVs for each biofluid. The protein lists were used for comparative analysis and gene ontology analysis.

**Supl. Figure 9**: The proteomic data for chitosan-isolated sEVs from the indicated biofluids were analyzed for the human protein data previously reported in Vesiclepedia. **a**: HEK-293 CCM, **b**: Urine, and **c**: Saliva.

**Supl. Figure 10**: EV purification by sucrose density gradient ultracentrifugation of HEK-293 CCM. The scUCF-purified EV pellet from HEK-293 CCM was bottom loaded on sucrose density gradient (90%-10%, bottom to top) and subjected to ultracentrifugation at 200,000 ×g for 16h following the published protocol (reference 32 of the manuscript). Following centrifugation six fractions (F1-F6) were collected from the top to the bottom of the gradient and transferred into new tubes. The EVs from the collected fractions were washed by adding 9 ml of PBS and pelleted by ultracentrifugation at 138,000 ×g for 2h. The density gradient-purified EVs were subjected to Western blot analyses for canonical EV markers CD63 and CD9 to identify EV-enriched fractions. The EVs purified by scUCF and chitosan were used as reference samples in this experiment. Fractions F2, F3, and F4 were found to be enriched with EVs.

**Supl. Figure 11**: Comparative proteomic analysis of chitosan-isolated EVs and sucrose density gradient (DG)-purified EVs from HEK-293 CCM. **Bottom Panel**: FunRich-based ontology for cellular component for the proteins list as designated. Match percentages are shown for the top 5 cellular components based on p-value.


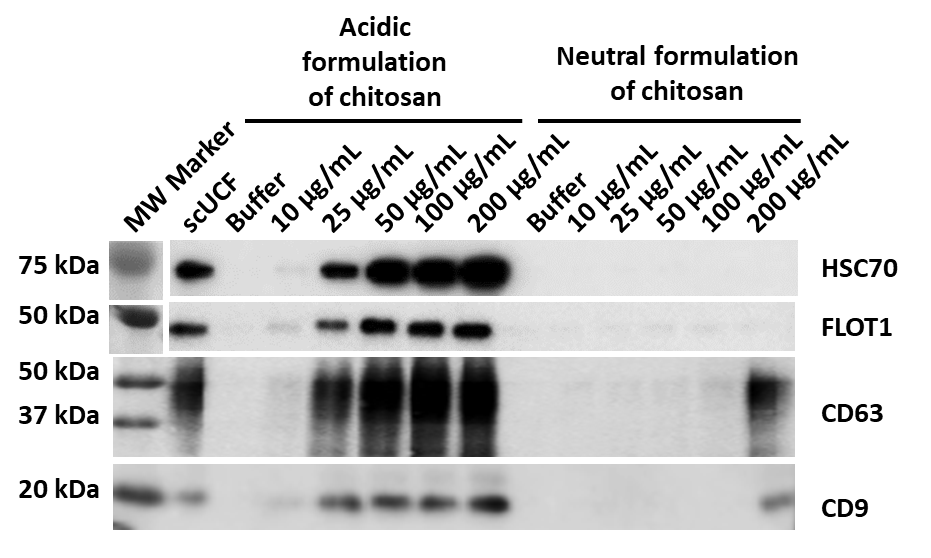


**Supplementary Figure 1**

**Supplementary Figure 2**


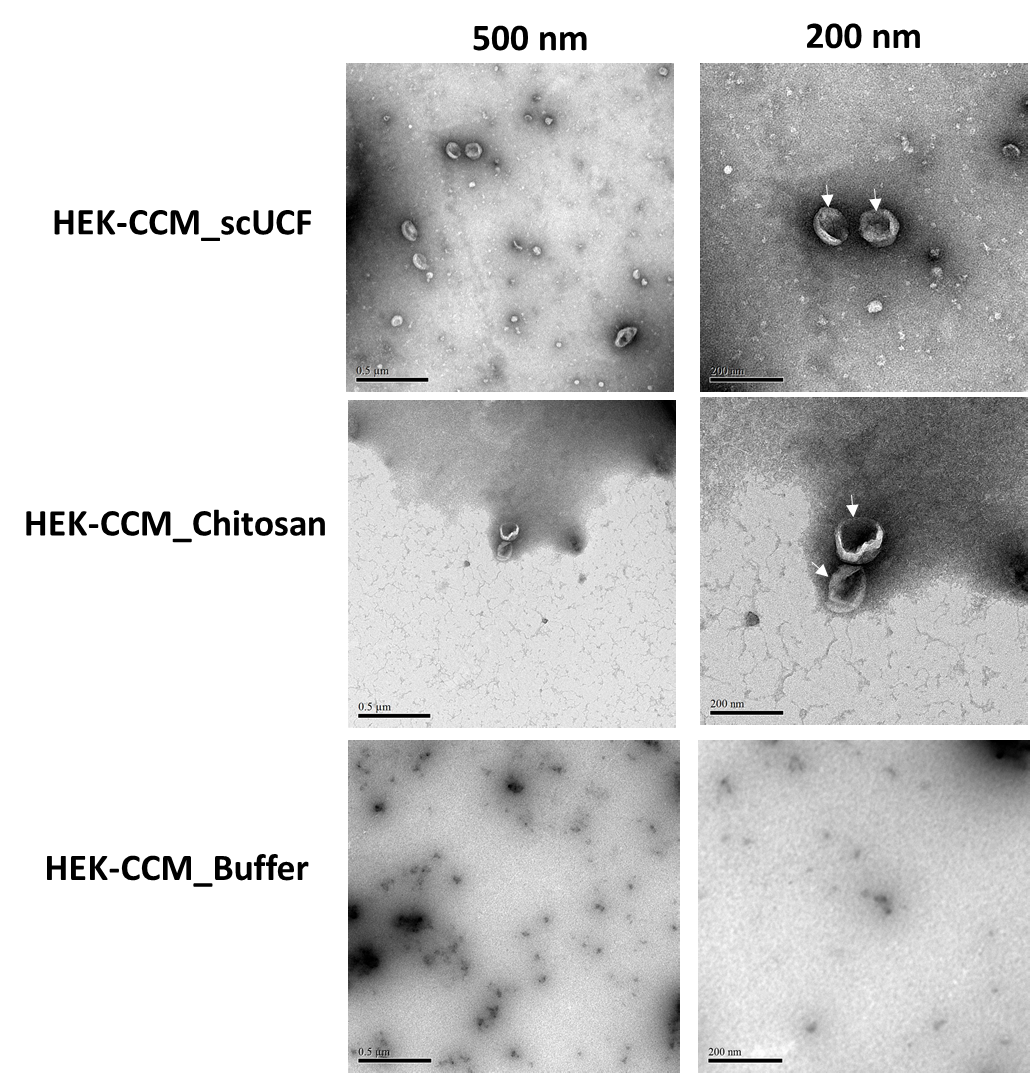


**Supplementary Figure 3**


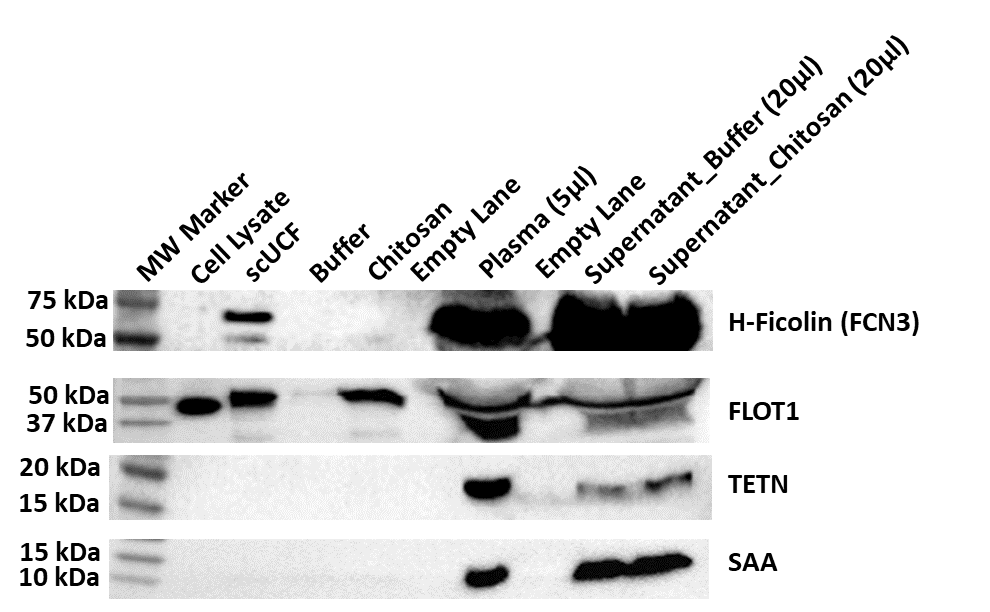


**Supplementary Figure 4**


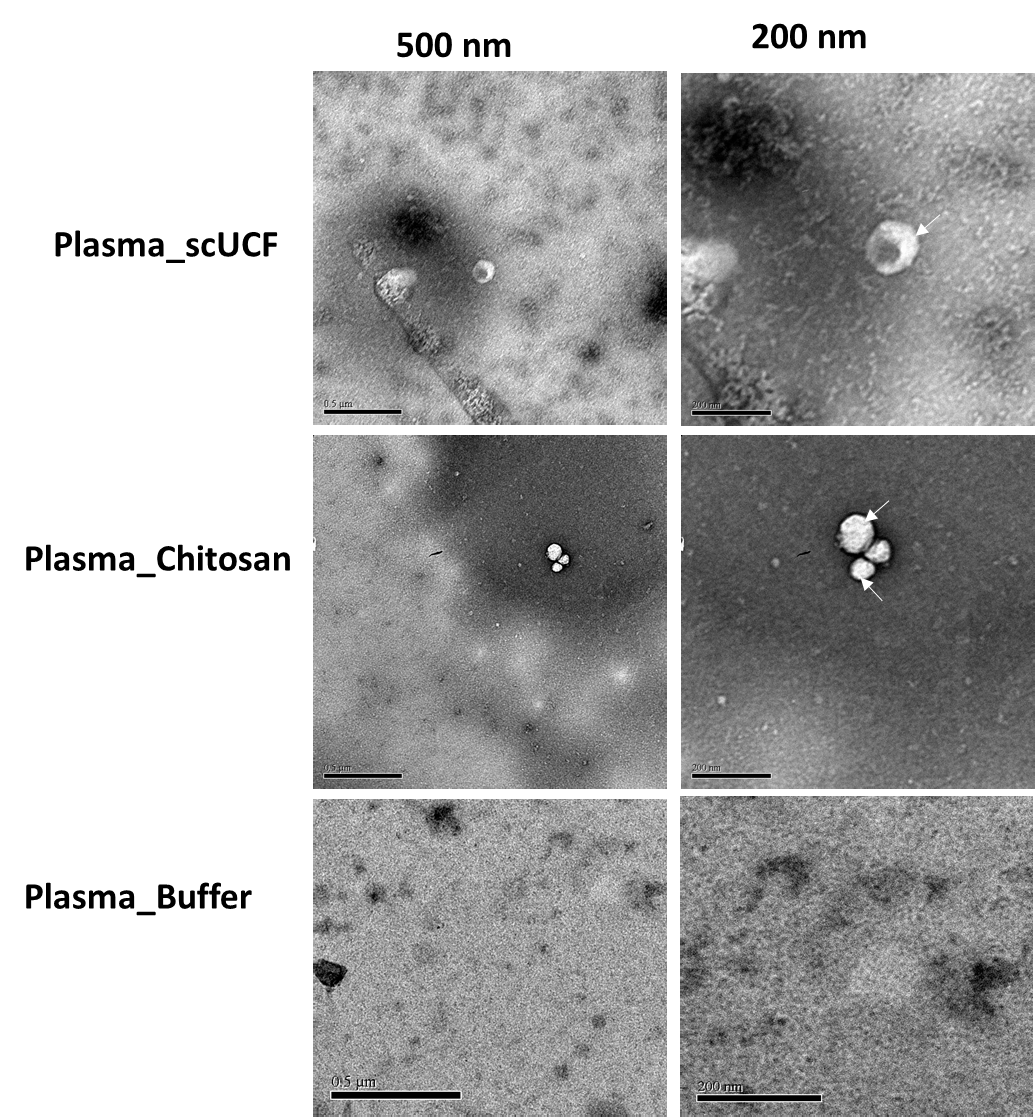


**Supplementary Figure 5**


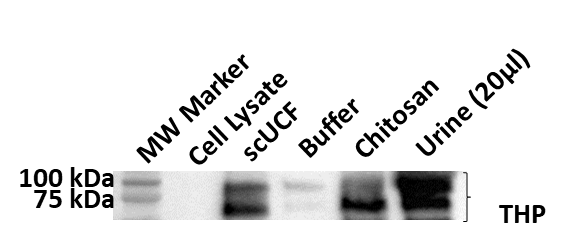


**Supplementary Figure 6**


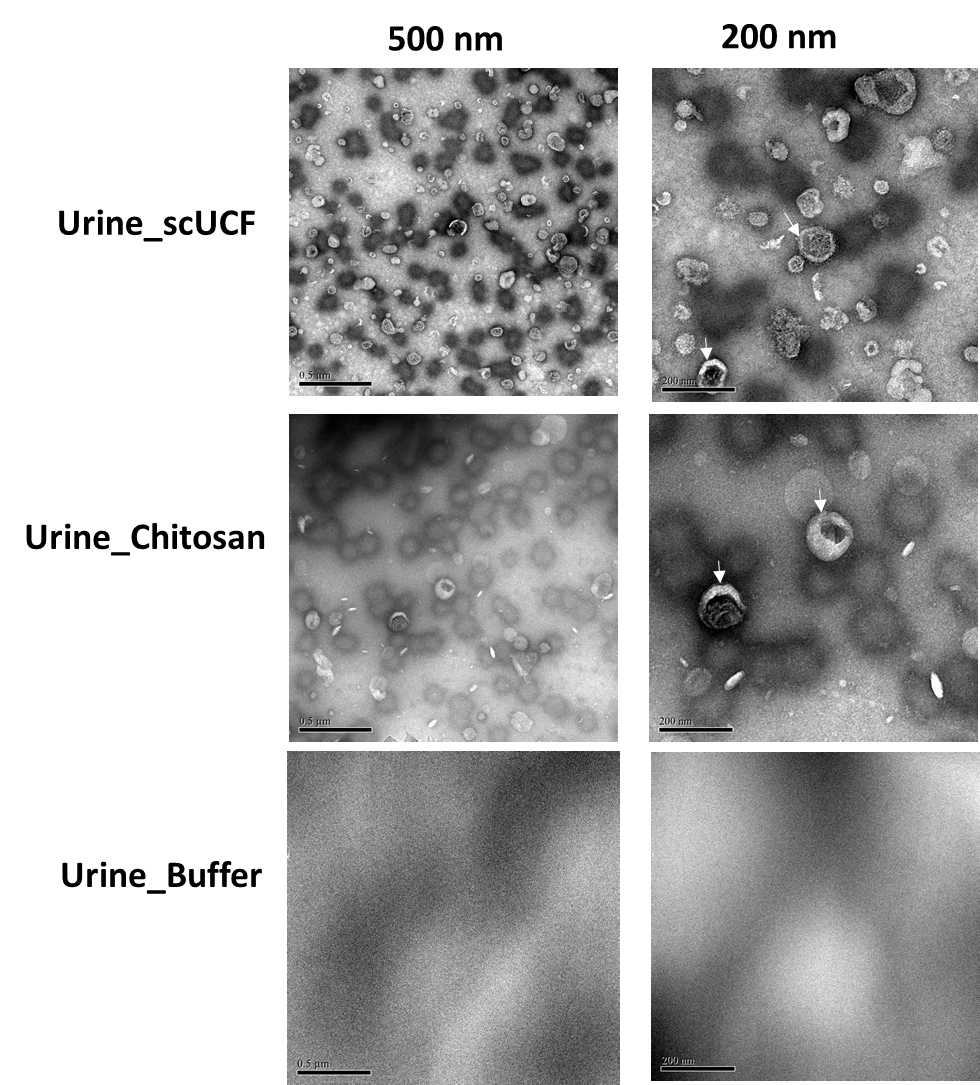


**Supplementary Figure 7**


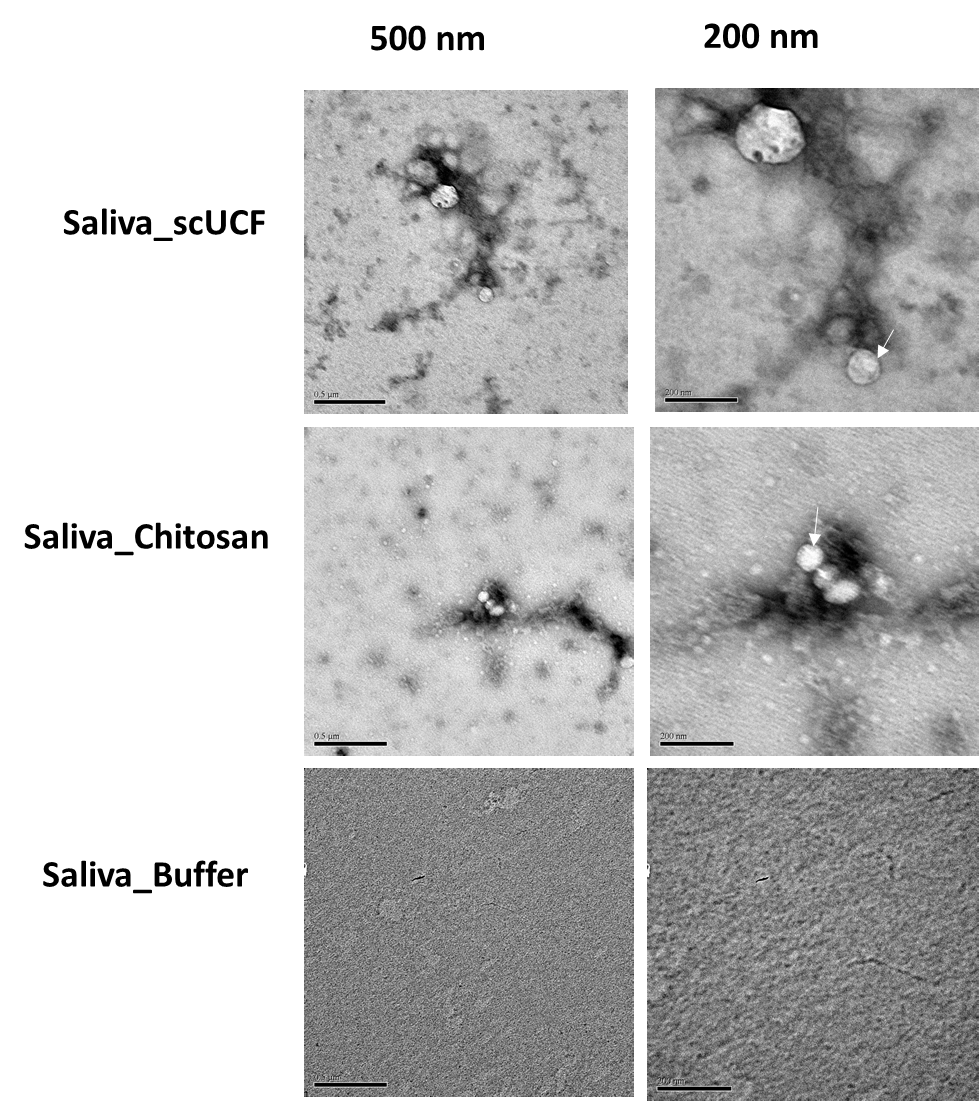


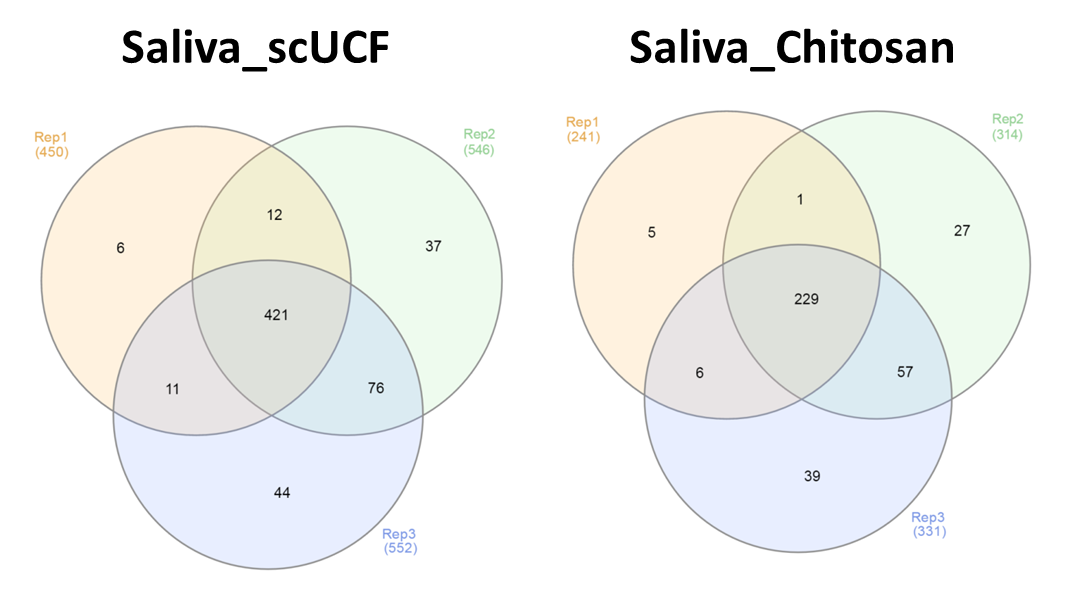

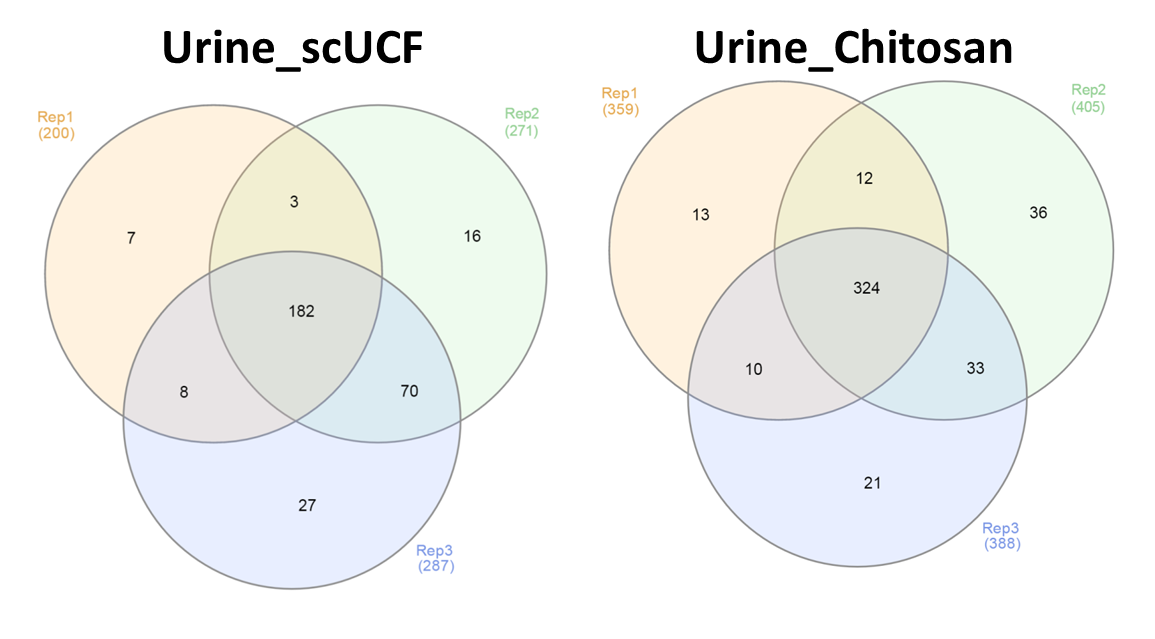

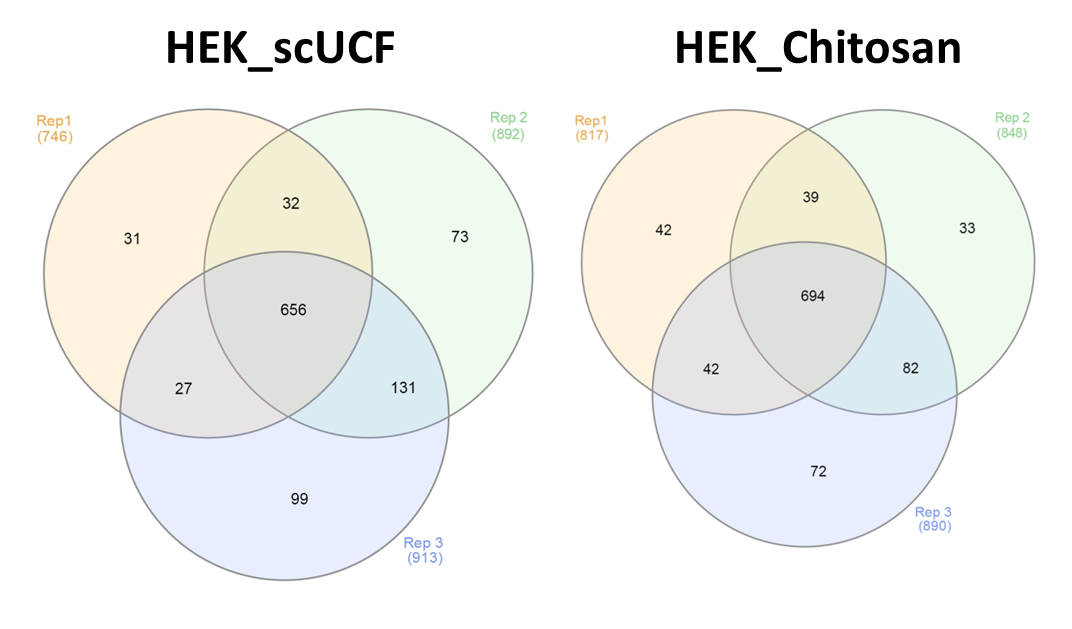


**Supplementary Figure 8**

**Supplementary Figure 9**


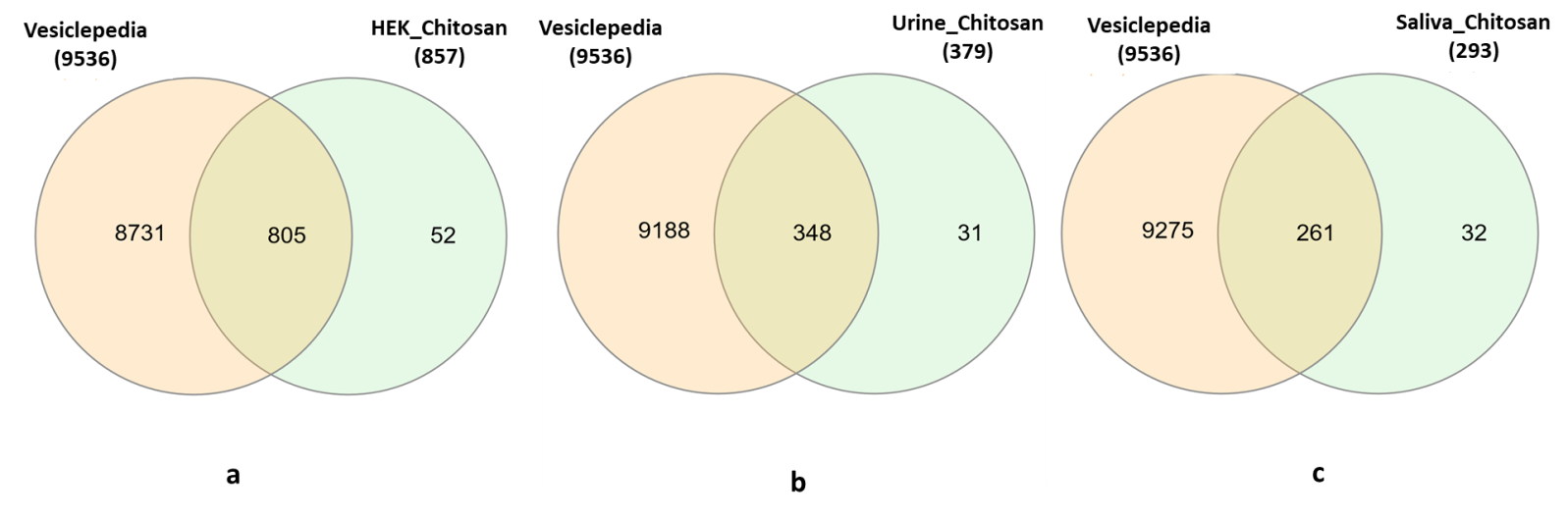


**Supplementary Figure 10**


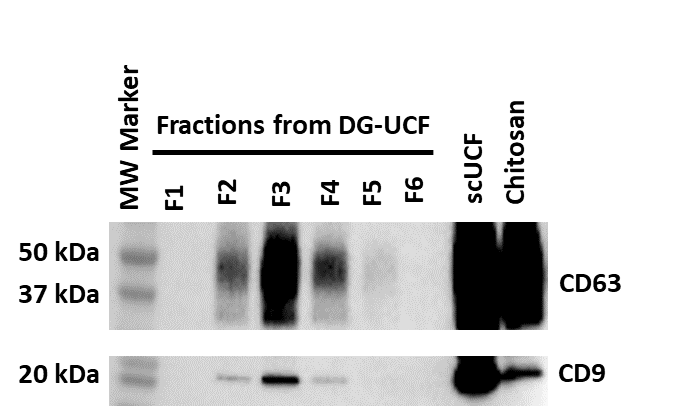


**Supplementary Figure 11**


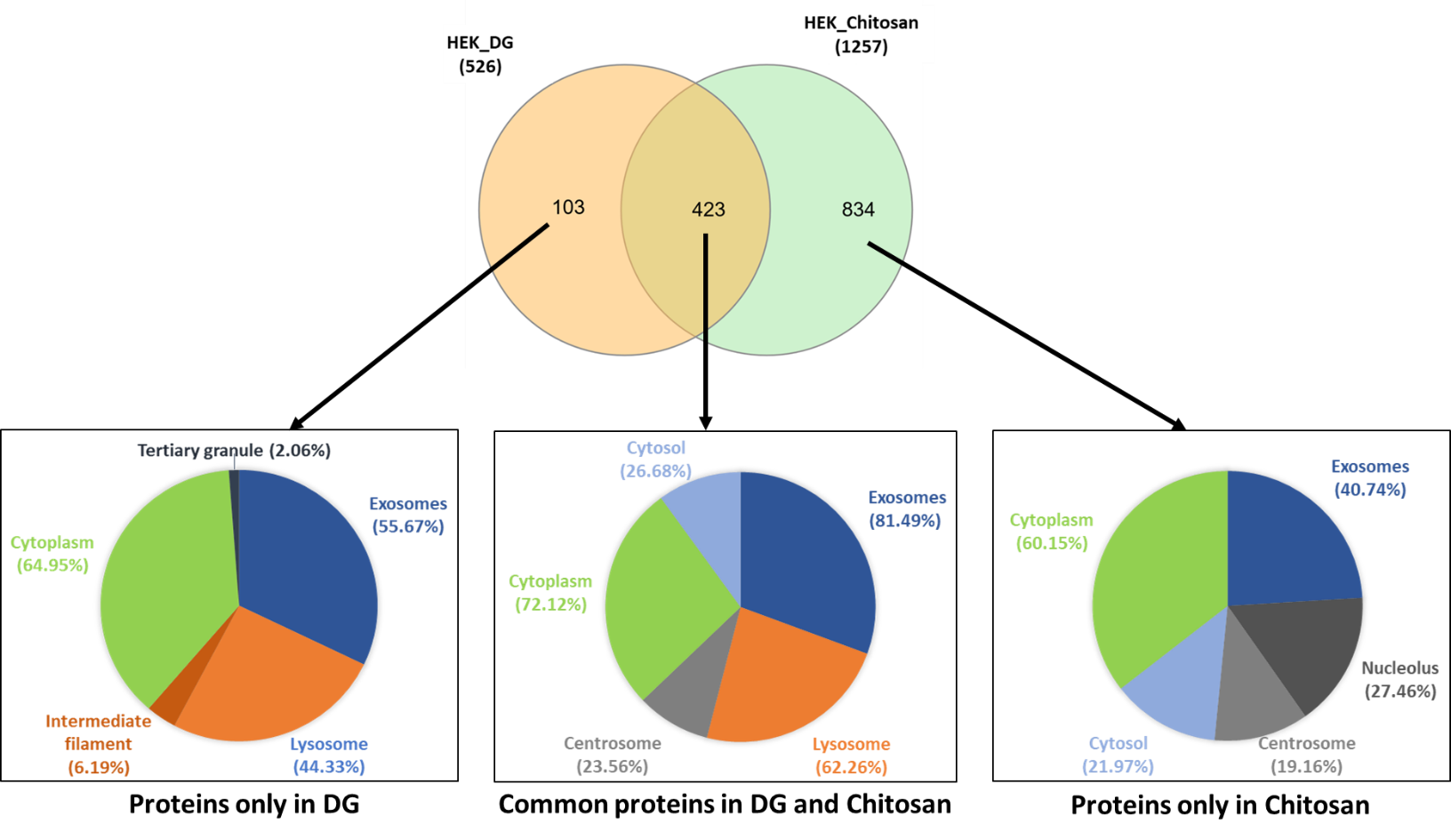

Supplement: Supplementary file 1 — Supporting Information [file JEV2-10-e12138-s002.docx]
